# Development of GFP-expressing infectious clones for PRRSV using TAR cloning for antiviral drug screening

**DOI:** 10.1038/s44298-025-00148-3

**Published:** 2025-09-05

**Authors:** Minze Zhang, Bang Qian, Dusan Kunec, Michael Veit

**Affiliations:** Faculty of Veterinary Medicine, Institute of Virology, Freie Universität Berlin, Berlin, Germany

**Keywords:** Virology, Biological techniques

## Abstract

Porcine reproductive and respiratory syndrome virus (PRRSV), an *Arteriviridae* family enveloped RNA virus, is a major swine pathogen. Using yeast transformation-associated recombination (TAR) cloning, we efficiently generated infectious PRRSV and GFP-expressing clones, identifying transcription-regulating sequences as essential for stable foreign gene expression. Screening SARS-CoV-2 antivirals showed potent inhibition by the multitarget drug ribavirin, the polymerase inhibitors remdesivir and its metabolite GS-441524. Molnupiravir, targeting the polymerase by a different mechanism, showed reduced efficacy against PRRSV, while the protease inhibitor GC376 was ineffective. The AlphaFold-predicted structure of the PRRSV polymerase revealed conserved catalytic architecture with the SARS-CoV-2 polymerases, explaining cross-family inhibitor activity. In contrast, structural divergence in proteases correlated with GC376’s inefficacy. These findings underscore the utility of the TAR cloning for arterivirus engineering, with potential applications in vector vaccine development.

## Introduction

Porcine reproductive and respiratory syndrome virus (PRRSV) is a major pathogen affecting the global pig industry, causing significant economic losses through reproductive failures in sows and severe respiratory diseases in piglets^1^. PRRSV belongs to the *Arteriviridae* family and is classified into two species: *Betaarterivirus suid* 1 (PRRSV-1, prototype strain Lelystad) and *Betaarterivirus suid 2* (PRRSV-2, prototype strain VR-2332)^2^. Since its discovery, PRRSV has spread worldwide, rapidly diversifying and leading to the emergence of highly pathogenic variants, that pose ongoing challenges to the swine industry^3–7^. In pigs, PRRSV infects alveolar macrophages and in vitro it replicates in porcine alveolar macrophages (PAMs) and in the MARC-145 cell line.

PRRSV is an enveloped, positive-sense RNA virus with a genome of ~15 kb. Its genome structure includes a 5’ untranslated region (UTR) containing the leader sequence, 11 open reading frames (ORFs), and a 3**′**UTR followed by a poly(A) tail. The 5**′** terminal two-thirds of the genome contain ORF1a and ORF1ab, which encode at least 14 nonstructural proteins (nsp) essential for viral replication and transcription^8,9^. These include the RNA-directed RNA polymerase (nsp9), and two proteases—papain-like cysteine protease (nsp2), and serine protease (nsp4)—which are crucial for processing the polyprotein precursors encoded by ORF1a and ORF1ab. The 3**′** terminal part of the genome encodes seven structural proteins: Gp2, E, Gp3, Gp4, ORF5a, Gp5, M, and N. Gp5 forms a disulfide-linked complex with the membrane protein (M), and along with the nucleocapsid protein (N), constitutes the major virion components essential for virus budding. The heterotrimeric complex formed by Gp2, Gp3, and Gp4, likely in conjunction with the small envelope protein E, is crucial for cell entry^10–12^.

These structural proteins are translated from subgenomic mRNAs, which are generated by a discontinuous transcription mechanism. This process produces a nested set of 3′ coterminal transcripts that contain a common 5**′** leader sequence derived from the genomic 5**′** terminus^13,14^. During negative-strand RNA synthesis, the viral polymerase may perform template-switching events at body TRS (transcription-regulating sequence), enabling it to skip to the leader TRS and generate subgenomic RNAs. These negative-strand subgenomic RNA are subsequently copied into positive-strand mRNA for protein synthesis. Although the mRNA contains several open reading frames, only the first one is translated by the ribosome^15,16^.

TRS play a pivotal role in subgenomic RNA synthesis for PRRSV. The leader TRS, with the sequence [UUAACC], is conserved across all PRRSV-1 and PRSV-2 strains^17^. In contrast, the body TRS exhibit sequence variability between PRRSV-1 and PRRSV-2 strains. PRRSV-2 strains typically feature body TRS with the consensus sequence [U/A/G][U/A/G][A/C][A/G][C/U]C, while PRRSV-1 strains generally follow the pattern U[A/U/C][A/G][A/C]CC. However, which specific TRS region is optimal to drive exogenous gene expression in PRRSV remains largely unexplored^18–21^.

The distance between body TRS sites and their associated start codons varies considerably between PRRSV-1 and PRRSV-2 strains. In PRRSV-2, this distance ranges from 16 to 229 nucleotides, with TRS6 maintaining the shortest distance of 16 nucleotides to its corresponding ORF6. However, the functional implications of these differences (e.g., translation efficiency, ribosomal accessibility) remain unknown. Notably, TRS6 has been demonstrated to be highly effective in regulating GFP expression without compromising PRRSV-2 replication when GFP was inserted as an independent cassette driven by various body TRS at the ORF7/3**′**UTR junction^22^. PRRSV-1 strains, on the other hand, exhibit a more compact arrangement, with distances between body TRS sites and downstream start codons ranging from 9 to 83 nucleotides^19^. This diversity in body TRS, along with their positioning relative to downstream start codons, significantly influences the expression levels of the corresponding proteins.

Due to similarities in the genome organization and replication strategy, *Arteriviridae* are grouped in the order *Nidovirales* together with the family *Coronaviridae*. Consequently, they share proteins with similar structure and function. Among the structural proteins, M of coronaviruses is structurally similar to Gp5/M of PRRSV^23^. A protein with a structure similar to the spike of coronaviruses is missing in arteriviruses, but the Gp2/3/4 trimer might fulfil its function during cell entry. Key similarities in the non-structural protein include viral proteases, including papain-like (nsp2 in arteriviruses and nsp3 in coronaviruses) and chymotrypsin-like proteases (nsp4 in arteriviruses and nsp5 in coronaviruses), which are utilized for polyprotein processing. The RNA-dependent RNA polymerase (RdRp, nsp9 in arteriviruses and nsp12 in coronaviruses), is essential for genome replication and transcription. Whereas structures of coronavirus nsp12 at several stages of replication have been resolved^24,25^, no structural information is available for nsp9 of any arterivirus. Based on these similarities, antiviral drugs developed for SARS-CoV-2 treatment may also have potential to inhibit PRRSV replication.

Reverse genetics has become an invaluable tool for studying PRRSV, allowing researchers to introduce precise modifications at specific sites or regions of the viral genome. This technique enables the creation of modified infectious viruses, facilitating investigations into virus replication, pathogenesis, and the functions of individual viral proteins. Additionally, it has proven crucial for developing viruses as vectors for vaccines^26^. Conventional approaches for rescuing infectious PRRSV typically involve either DNA-based or RNA-based strategies. The DNA-based method entails transfecting cells directly with a plasmid or a bacterial artificial chromosome (BAC) containing a full-length PRRSV cDNA clone under the control of a eukaryotic polymerase II promoter, such as the human cytomegalovirus (CMV) immediate-early promoter^27,28^. Alternatively, the RNA-based approach generates in vitro transcribed viral RNA transcript from cDNA, often maintained in a low-copy-number plasmid or a BAC. While BACs can serve as cloning vectors in both scenarios, the fundamental distinction lies in whether RNA or DNA is introduced to permissive cells to initiate the virus rescue^29,30^. Wang et al. introduced a DNA-launched PRRSV system utilizing a BAC, which facilitates mutagenesis^31^.

However, these conventional methods present several challenges. They often require multiple cloning steps dependent on unique restriction enzyme sites and involve cumbersome screening procedures. The construction and modification of full-length cDNA clones are laborious and time-consuming, impeding the rapid development of infectious clones for new virus strains. Furthermore, genomic instability is a well-recognized challenge in assembling and maintaining large viral cDNA clones in bacteria. Although this concern has been specifically described for coronaviruses with their larger genomes, similar instability issues in *Escherichia coli* have been reported for arteriviruses like PRRSV^32^.

Recently, a novel approach for rescuing infectious PRRSV particles called the “Infectious-Subgenomic Amplicons” (ISA) method was introduced^32^. The ISA method utilizes four to five overlapping DNA fragments spanning the entire PRRSV genome, which are directly transfected into a co-culture of BHK-21 and MARC-145 cells without the need for fragment ligation. This approach eliminates the time-consuming cloning procedures typically associated with conventional reverse genetics systems. Despite its advantages, the ISA method presents several limitations that warrant consideration. The molecular mechanisms underlying virus rescue using this approach remain unclear, particularly the processes of DNA fragment ligation and subsequent transcription within eukaryotic cells. Additionally, viral populations generated using the ISA method tend to exhibit greater genetic diversity compared to those derived from complete infectious clones, potentially complicating the selection of desired mutants. Furthermore, viruses rescued via the ISA method often require more serial passages in MARC-145 cells to achieve sufficient titers compared to those generated from complete infectious clone^33,34^.

In this study, we employed a yeast-based transformation-associated recombination (TAR) cloning system to construct infectious cDNA clones of both PRRSV-1 and PRRSV-2^35,36^. This innovative platform, previously successfully applied to SARS-CoV-2 and feline infectious peritonitis viruses, significantly accelerates the process from cDNA clone construction to virus rescue, completing it within one week. The TAR cloning system offers a versatile and efficient alternative for rapidly constructing infectious clones of PRRSV strains, accommodating DNA fragments from diverse sources, including synthetic DNA and PCR products from newly isolated field strains^35,37,38^. Leveraging this system, we further engineered infectious clones of both PRRSV-1 and PRRSV-2 to generate GFP-expressing recombinant viruses. We used these fluorescent reporter viruses to investigate the effect of various antiviral drugs that are known to inhibit replication of SARS-CoV-2 and other RNA viruses. Our approach not only streamlines the creation of infectious PRRSV clones but also enhances their utility in both basic research and applied virology, potentially accelerating the development of novel vaccines and therapeutics against this economically significant pathogen.

## Results

### Construction of infectious clones of PRRSV-1 and PRRSV-2 by TAR cloning

**Fig. 1:**
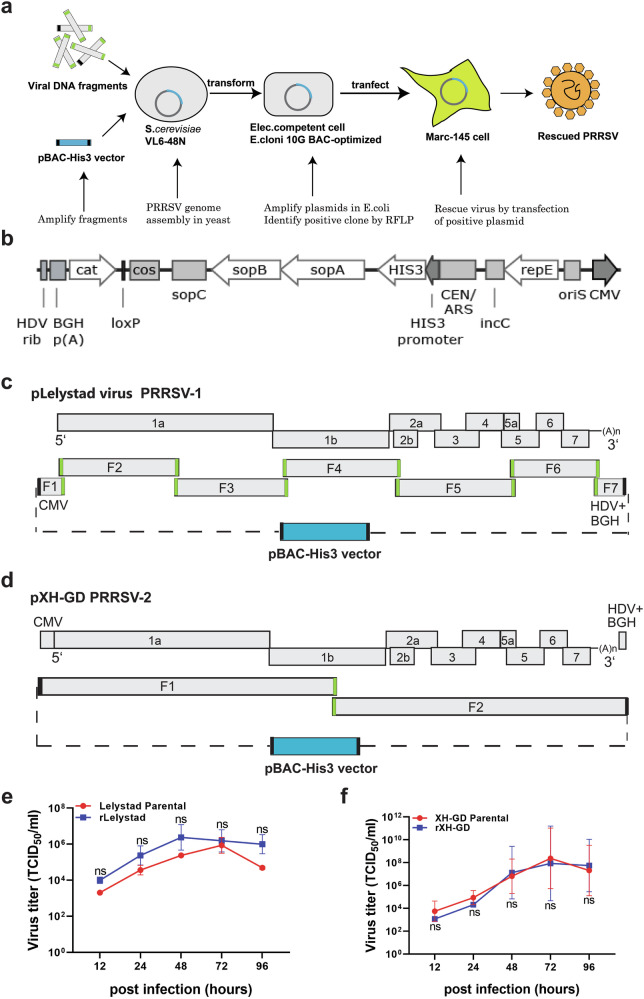
Strategy for the construction of infectious cDNA cones of PRRSV-1 and PRRSV-2 by TAR cloning in yeast. **a** Workflow illustrating the assembly of infectious cDNA clones in yeast and subsequent rescue of recombinant viruses. **b** Genetic map of the TAR vector pBAC-His3. The backbone is derived from a bacterial artificial chromosome (BAC), which ensures stable single-copy maintenance in *E. coli*. It also incorporates yeast artificial chromosome (YAC) elements which enable replication and selection in yeast: a centromere (CEN), autonomously replicating sequence (ARS), and HIS3 selectable marker. The vector contains regulatory elements essential for viral RNA synthesis in mammalian cells: a human cytomegalovirus immediate-early promoter (CMV), hepatitis delta virus ribozyme (HDV rib), and bovine growth hormone polyadenylation signal (BGH p(A)). The CMV promoter drives efficient transcription of the viral genome, while the HDV ribozyme and BGH polyadenylation signal ensure precise 3′-end processing and mRNA termination. Together, these elements produce 5′-capped and 3′-polyadenylated viral RNA transcripts that mimic native PRRSV genomic RNA. **c**, **d** Schematic overview of the construction of infectious clones for XH-GD (PRRSV-2) and Lelystad virus (PRRSV-1), respectively. Upper parts show genome organization of PRRSV-2 (**c**) and PRRSV-1 (**d**). Lower parts show PCR-amplified subgenomic overlapping fragments used for the assembly of infectious clones alongside with the TAR vector pBAC-His3. Green regions indicate overlapping sequences. **e**, **f** Growth kinetics of parental and recombinant viruses (rXH-GD and rLelystad). MARC-145 cells were infected with either the parental or recombinant virus (MOI = 0.01 for XH-GD virus, and MOI = 0.1 for Lelystad virus). Cell culture media were collected at the indicated time points, and virus titers were determined by TCID_50_ assay. Data represent geometric mean titers ±SD from three independent experiments. Statistical analysis was performed using two-way ANOVA with Bonferroni post-test. No significant (ns) differences in viral titers were detected between the parental and recombinant viruses at any time point.

We constructed infectious cDNA clones of PRRSV-1 (strain Lelystad) and PRRSV-2 (strain XH-GD) using TAR cloning in *Saccharomyces cerevisiae* (Fig. 1a). To achieve this, we first amplified viral genomes as overlapping fragments via PCR using sequence-specific primers (Table S1). These fragments were subsequently co-transformed into *S. cerevisiae* along with the TAR vector pBAC-His3, a hybrid shuttle vector capable of propagation in both *E. coli* and *S. cerevisiae* (Fig. 1b). Homologous recombination in yeast facilitated the accurate assembly of full-length viral genomes within the vector backbone, generating complete infectious cDNA clones.

To construct the infectious clone of PRRSV-1, viral RNA was isolated form infected cells, reverse transcribed into cDNA, and the complete viral genome was amplified as five overlapping fragments (fragments 2–6). Two short synthetic fragments (fragments 1 and 7), corresponding to the vector–virus junctions (Fig. 1c), were designed to facilitate homologous recombination between the viral genome and the pBAC-His3 vector. For PRRSV-2, the viral genome was amplified form an existing plasmid containing the XH-GD genome as two overlapping fragments (Fig. 1d).

TAR cloning was performed as previously described^39^, and each assembly yielded hundreds of yeast colonies on selective agar plates. DNA was extracted from several randomly selected yeast clones, and transformed into BAC-optimized *E. coli*. BAC DNA was subsequently isolated from *E. coli* clones and the correctness of the assembled PRRSV-1 (pBAC-His3-LV) and PRRSV-2 (pBAC-His3-XH-GD) constructs was assessed by restriction fragment length polymorphism (RFLP) analysis. This assay revealed that most of the tested clones exhibited the expected restriction profiles, indicating that the yeast-based assembly was highly efficient. Finally, several clones with the correct restriction digestion profiles were further validated by whole-genome nanopore sequencing.

To rescue infectious viruses, the PRRSV-1 and PRRSV-2 BAC DNA was transfected into HEK 293T cells, which are known for their high transfection efficiency and ability to produce infectious PRRSV particles. Three days after transfection, cell culture media from transfected cells were used to infect MARC-145 cells, yielding recombinant viruses rLV and rXH-GD. Characteristic cytopathic effect (CPE) was observed within 2–3 days post infection, and the presence of viral infection was confirmed via immunofluorescence using monoclonal antibodies targeting the PRRSV nucleocapsid protein (Supplementary Fig. 1). Growth kinetics analysis showed that the recombinant viruses exhibited replication dynamics similar to their respective parental strains, reaching peak titers of ~10^6^ TCID_50_/mL for PRRSV-1 and 10^8^ TCID_50_/mL for PRRSV-2 between 48- and 72-h post infection (Fig. 1e, f).

### Construction and characterization of GFP reporter PRRSV-1 and PRRSV-2

**Fig. 2:**
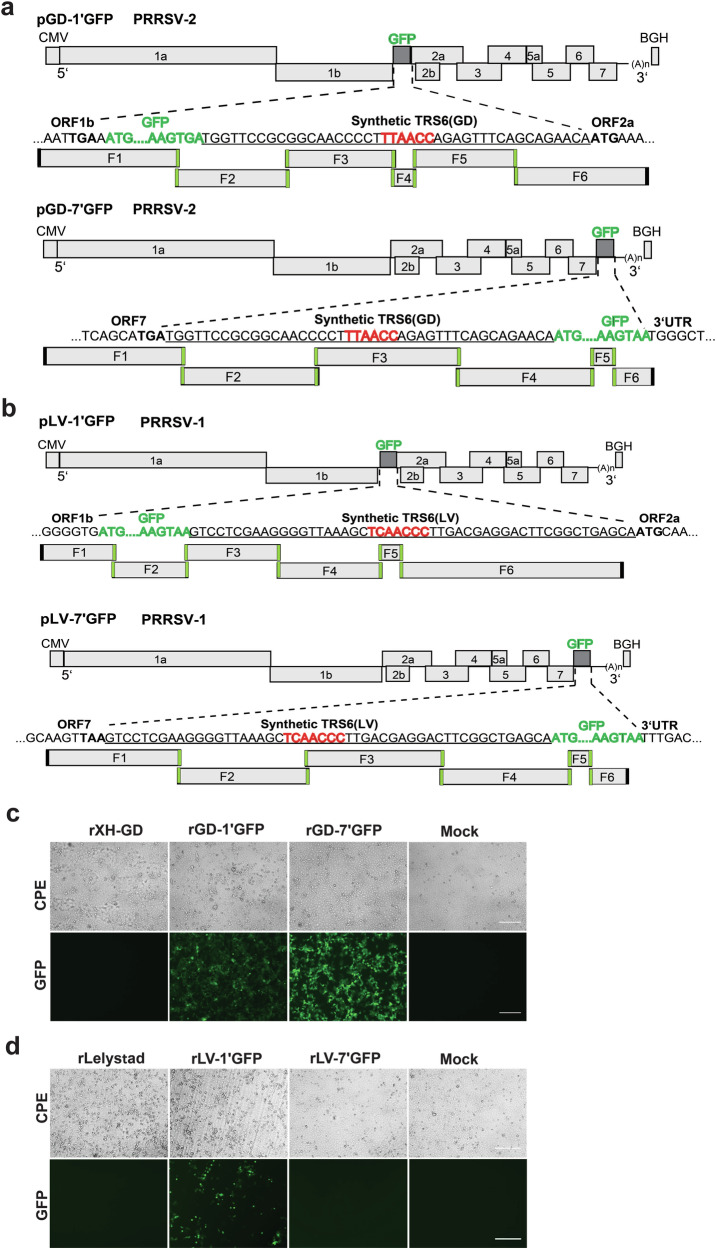
Construction and rescue of reporter GFP viruses by TAR cloning. Schematic overview of the construction of infectious clones pGD-1’GFP and pGD-7’GFP (**a**), and pLV-1’GFP and pLV-7’GFP (**b**) using TAR cloning. The diagrams illustrate the genomic organization of the resulting clones and the overlapping fragments used for their assembly. Overlapping sequences are highlighted in green. The GFP ORF was inserted at two genomic locations: between ORF1 and ORF2, and between ORF7 and the 3′UTR. GFP sequences are shown in bold green. Sequences containing transcription regulatory sequence 6 (TRS6) are underlined; the core TRS6 motif is shown in bold red. Start codons of ORF2a (ATG) and stop codons of ORF7 (TGA or TAA) are shown in bold. In constructs with GFP inserted between ORF1 and ORF2, TRS6 is located downstream of GFP and regulates the expression of ORF2 (GP2), while GFP expression is controlled by native TRS2. For constructs with GFP inserted between ORF7 and the 3′UTR, TRS6 is positioned upstream of the GFP ORF to drive its expression. **c**, **d** Validation of GFP reporter virus rescue. DNA from the reporter clones was transfected into HEK 293T cells. After 72 h, cell culture media were harvested and used to infect MARC-145 cells. Infected cells were examined 48–72 h post infection for characteristic cytopathic effects and GFP fluorescence. Mock: uninfected control. Scale bar: 100 μm.

GFP reporter constructs of PRRSV-1 and PRRSV-2 were constructed using the same TAR cloning strategy described above. The GFP expression cassette was inserted at two genomic positions: between the ORF1 and ORF2, and between the ORF7 and the 3**′**UTR. To generate viruses expressing GPF from the ORF1-ORF2a location, the GFP ORF was inserted immediately downstream of ORF1. In this configuration, TRS2, located within ORF1 and approximately 25 nucleotides upstream of the ORF2a start codon was repurposed to drive the GFP mRNA synthesis. To ensure ORF2a expression, a copy of a native sequence containing TRS6 was introduced immediately downstream of the GFP ORF (Fig. 2a, b; see also Table 1 for a summary of the constructs). TRS6 was selected to drive ORF2 expression because it has previously been shown to mediate robust expression of heterologous genes in PRRSV-2^31,40,41^. In PRRSV-1, the duplicated sequence was 50 bp long and included the TRS6 core motif ‘TCAACC’, whereas in PRRSV-2, the duplicated sequence was 42 bp long and contained the TRS6 core motif ‘TTAACC’. These constructs were designated pLV-1’GFP (PRRSV-1) and pGD-1’GFP (PRRSV-2). To ensure efficient GFP expression from the ORF7-3**′**UTR region, the same TRS6-containing sequences were placed immediately upstream of the GFP ORF. The resulting bacterial constructs were designated pLV-7’GFP (PRRSV-1) and pGD-7’GFP (PRRSV-2), respectively. The GFP reporter constructs were assembled from six overlapping fragments: five corresponding to the complete BAC sequence, and a sixth fragment contained the GFP ORF and the associated TRS6 sequence (Fig. 2a, b).

**Table 1:** GFP-expressing PRRSV-1 and PRRSV-2 generated by TAR cloning

Virus	Virus rescue	Gp2-expression driven by	GFP-expression driven by	GFP expression	Virus titers relative to parental virus	Stability of GFP expression
rGD-1’GFP	+	TRS6	TRS2	+/-	Comparable	8 passages
rGD-7’GFP	+	TRS2	TRS6	+	Comparable	8 passages
rLV-1’GFP	+	TRS6	TRS2	+	Slower growth	4 passages
rLV-1’GFP^TRS6(GD)^	+	TRS6 (GD)	TRS2	++	Comparable	19 passages
rLV-7’GFP	-	TRS2	TRS6	no	n.a.	
rLV-7’GFP^TRS6(GD)^	+	TRS2	TRS6 (GD)	+/-	n.a.	1 passage

Virus rescue: “+” indicates successful virus rescue; “–“ indicates failure to rescue. GFP expression: “++“ denotes stronger expression than the N protein, “+“ denotes expression similar to the N protein, “+/–“ indicates weaker expression than the N protein, and “n.a.” indicates not analyzed.

To rescue infectious viruses, DNA of each construct was transfected into HEK 293T cells and after 72 h, the harvested cell culture media were passaged to MARC-145 cells. Typical CPE was observed in cells infected with GD-1’GFP, GD-7’GFP and LV-1’GFP 3 days post-infection (dpi). Consistent with the onset of CPE, GFP expression was detected in cells infected with these three recombinant viruses, though expression was noticeably weaker in cells infected with LV-1’GFP (Fig. 2c, d). In contrast, no infectious virus was recovered from the pLV-7’GFP construct, suggesting that the ORF7–3**′**UTR insertion site may not be compatible with PRRSV-1 replication.

### Replacement of TRS6 sequence with its PRRSV-2 counterpart facilitated recovery of GFP reporter PRRSV-1

**Fig. 3:**
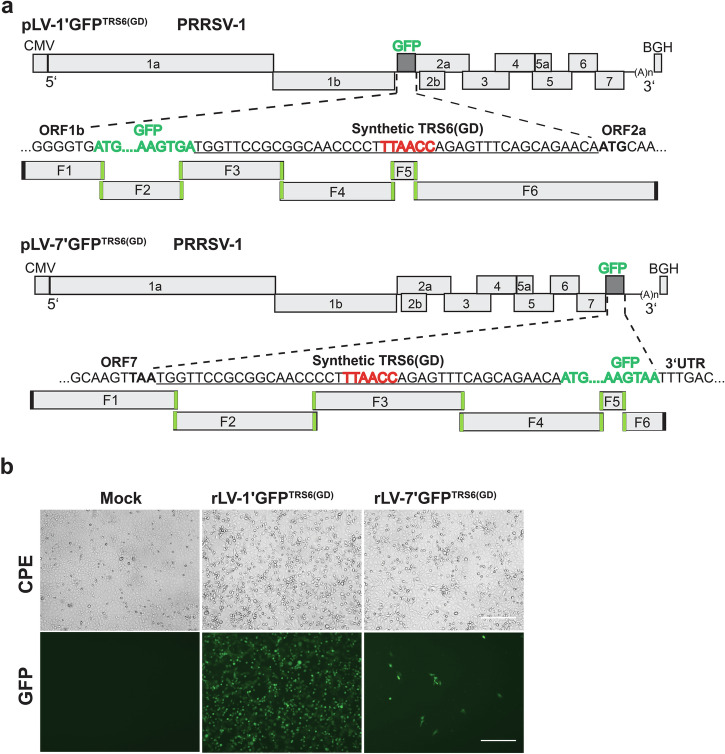
PRRSV-2-derived TRS6 improves rescue of GFP reporter PRRSV-1. **a** Schematic overview illustrating the construction of infectious clones pLV-1’GFP-TRS6(GD) and pLV-7’GFP-TRS6(GD). These constructs are analogous to pLV-1’GFP and pLV-7’GFP, but the original TRS6 was replaced by its counterpart from PRRSV-2 (strain XH-GD). The GFP reporter gene was inserted at two genomic positions in the Lelystad virus: between ORF1 and ORF2, and between ORF7 and the 3′UTR. Assembly of the GFP constructs was performed using five PCR-amplified fragments spanning the viral genome and the TAR vector (derived from pBAC-His3-Lelystad), along with one synthetic fragment encoding the GFP ORF and the TRS6. The TRS6 and flanking sequences are underlined and the core TRS6 motif is shown in bold red. Start codon of ORF2a (ATG) and stop codon of ORF7 (TAA) are also shown in bold. **b** Rescue of GFP reporter viruses rLV-1’GFP-TRS6(GD) and rLV-7’GFP-TRS6(GD) from the corresponding infectious clones in MARC-145 cells. Characteristic CPE was observed 48–72 h post infection, and GFP fluorescence was detected by fluorescence microscopy. Mock: uninfected control. Scale bar: 100 μm.

Since only one of the GFP reporter constructs based on the Lelystad virus could be rescued, and this virus showed only weak fluorescence, we tested whether GFP reporter mutants carrying PRRSV-2-derived TRS6 sequences might improve virus recovery and GFP expression. The resulting constructs, pLV-1’GFP-TRS6(GD) and pLV-7’GFP-TRS6(GD), used the PRRSV-2-derived TRS6 to regulate expression of ORF2a and GFP, respectively (Fig. 3a; see Table 1 for construct details). This modification not only facilitated the rescue of the recombinant rLV-7’GFP in MARC-145 cells but also enhanced GFP expression from the ORF1-ORF2a insertion site (Fig. 3b).

### GFP expression levels and growth characteristics of GFP reporter PRRSV

To assess GFP expression levels of PRRSV-1 and PRRSV-2 constructs, MARC-145 cells were infected at a low multiplicity of infection (MOI) of 0.01, using early passage virus stocks (passage 2). At 72 h after infection cells were lysed and proteins were analyzed by Western blotting using antibodies against GFP and the nucleocapsid (N) protein, with the latter serving as a marker of infection and a loading control.

**Fig. 4:**
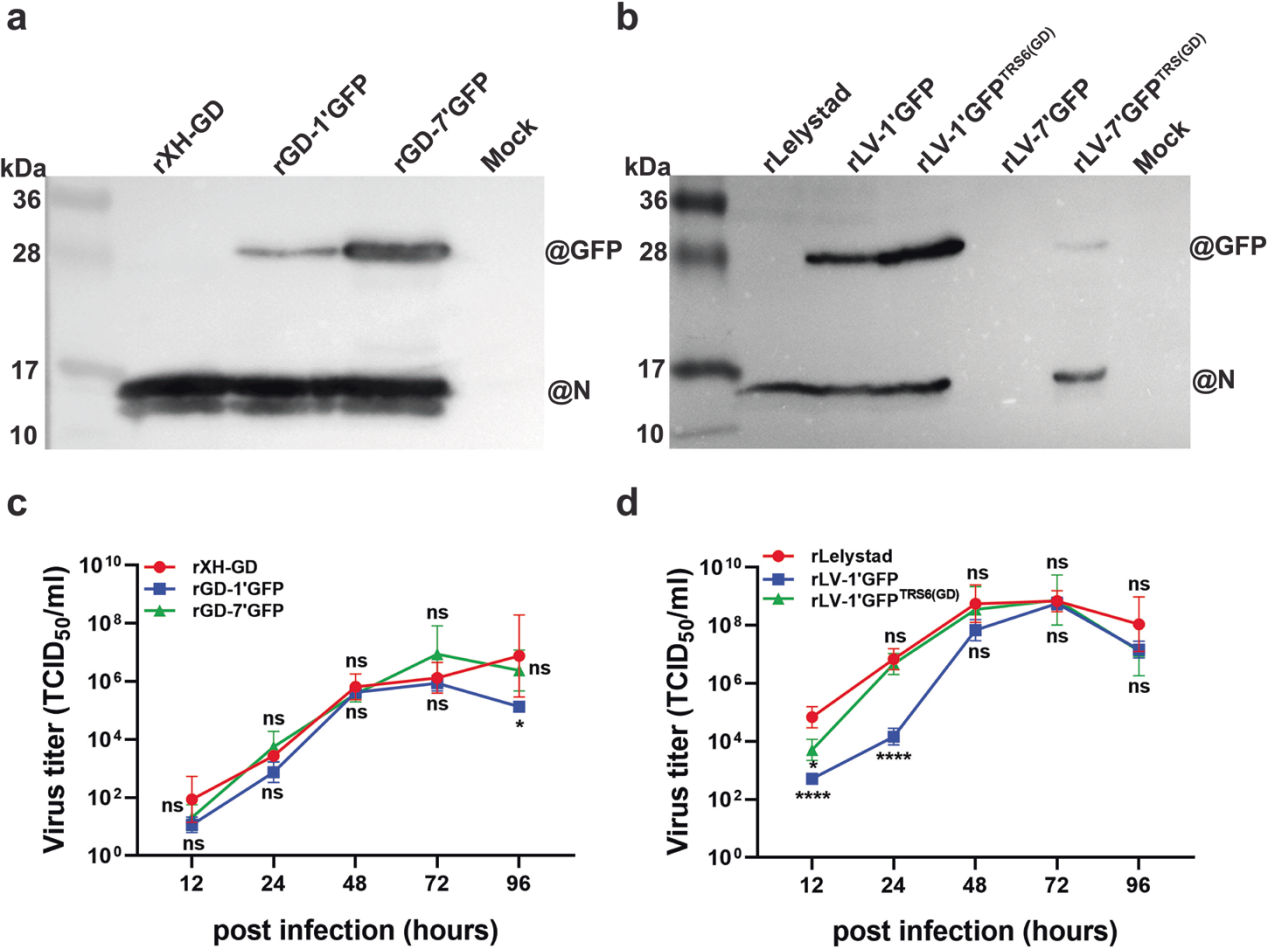
Characterization of GFP reporter viruses. **a**, **b** Analysis of GFP expression relative to viral N protein. MARC-145 cells were either mock-infected or infected with the indicated rescued viruses. Seventy-two hours after infection, cells were lysed and analyzed by Western blotting using an anti-GFP antibody. The same membranes were subsequently reprobed with antibodies specific to the N protein of PRRSV-1 or PRRSV-2. **c**, **d** Growth kinetics of GFP-expressing PRRSV-1 and PRRSV-2. MARC-145 cells were infected at a MOI of 0.01 (PRRSV-2) or 0.1 (PRRSV-1). Cell culture media were collected at the indicated time points, and virus titers were determined by TCID_50_ assay. Data represent geometric mean titers ±SD from three independent experiments. Asterisks denote statistically significant differences between wild-type (WT) and GFP-expressing viruses at the same time point (*P < 0.05, **P < 0.01, ***P < 0.001, ****P < 0.0001). Statistical analysis was performed using two-way ANOVA followed by Bonferroni post-test. Note: Absolute virus titers vary between experiments (compare with Fig. 1e, f). However, all viruses depicted within a single graph were analyzed in parallel.

From the two PRRSV-2 constructs, GD-1’GFP produced significantly less GFP than GD-7’GFP (Fig. 4a). In contrast, PRRSV-1 constructs displayed the opposite pattern: GFP expression was stronger when the GFP was inserted between ORF1 and ORF2, compared to the insertion at the ORF7-3**′**UTR site. Western blotting also confirmed that LV-1’GFP-TRS6(GD), carrying the TRS6 from PRRSV-2, produced more GFP that the corresponding construct with the native TRS6 from PRRSV-1. Similarly, TRS6 substitution enabled the successful rescue and GFP expression of LV-7’GFP-TRS6(GD). In contrast, the original rLV-7’GFP construct lacking the PRRSV-2-derived TRS6 showed no detectable expression of either GFP or N protein (Fig. 4b, Table 1).

The replication of GFP reporter viruses was evaluated in MARC-145 cells using multi-step growth kinetics with passage 2 virus stocks. Viral titers of the mutant viruses were compared to their respective parental strains, rescued from the corresponding infectious cDNA clones. For PRRSV-2, both GD-1’GFP and GD-7’GFP displayed growth kinetics similar to the parental rXH-GD virus up to 72 h post infection (hpi), reaching peak titers of ~10^6^ TCID_50_/mL. While rXH-GD titers continued to rise beyond 72 hpi, titers of the GFP-expressing variants declined slightly, suggesting reduced particle stability or diminished replication efficiency during later stages of infection (Fig. 4c).

In the case of PRRSV-1, the LV-1’GFP-TRS6(GD) construct followed a growth profile comparable to that of the parental rLV. Although rLV initially reached higher titers at early time points (12 and 24 hpi), both viruses reached similar titers at later stages of infection (Fig. 4d). The LV-7’GFP-TRS6(GD) construct was not included in the growth kinetics analysis due to the early loss of detectable GFP expression (see below).

Importantly, GFP expression from the PRRSV-2 GD-7’GFP and PRRSV-1 LV-1’GFP-TRS6(GD) constructs was also detectable by fluorescence microscopy in PAMs, the natural target cells of PRRSV (Supplementary Fig. 2).

### The stability of GFP expression in GFP reporter viruses in MARC-145 cells

The stability of GFP expression in the five rescued viruses was evaluated through serial passaging in MARC-145 cells. GFP expression in infected cells was monitored using fluorescence microscopy. All GFP reporter viruses demonstrated efficient replication, with significant CPE observed around 2–3 dpi at each passage. However, the number of passages where GFP expression was visible varied significantly between viruses.

PRRSV-2 constructs, GD-1’GFP and GD-7’GFP, exhibited stable GFP expression for up to eight passages. In contrast, PRRSV-1 recombinants showed varying levels of GFP expression stability. The LV-1’GFP virus maintained GFP expression for four passages before the GFP signal got lost. Substitution of the TRS6 element with its PRRSV-2 counterpart in LV-1’GFP-TRS6(GD) markedly enhanced GFP stability, with fluorescence maintained for up to 19 passages in MARC-145 cells. Conversly, LV-7’GFP-TRS6(GD) rapidly lost its ability to produce GFP. During early passages, only a few infected cells exhibited GFP fluorescence, which was no longer detectable by passage 3 (Supplementary Fig. 3, Table 1). These findings highlight the critical role of both the insertion site and TRS sequence selection in maintaining foreign gene expression during serial passaging.

### Antiviral assay using GFP reporter PRRSV-1 and PRRSV-2

Antiviral assays were conducted in MARC-145 cells, the only established cell line permissive for PRRSV, providing a consistent, reproducible, and practical system for high-throughput testing^42^. Although PAMs, the natural target cells for PRRSV, better reflect the in vivo environment, their limited availability, handling challenges, batch variability from donor animals, limited lifespan in culture, and less distinct cytopathic effect (CPE) make them less feasible for antiviral studies. We utilized PRRSV-1 LV-1’GFP-TRS6(GD) and PRRSV-2 GD-7’GFP viruses, which exhibit strong and stable GFP expression, to evaluate the antiviral efficacy of five drugs known to efficiently inhibit replication of SARS-CoV-2 in cell culture^43–49^. Four of them (remdesivir, its main metabolite GS-441524, molnupiravir [EIDD-2801], and ribavirin) are nucleoside analogues that target the viral RdRp, whereas GC376 is an inhibitor of the main protease 3CL^pro^ (M^Pro^). (see Supplementary Fig. 4 for structural formulas).

**Fig. 5:**
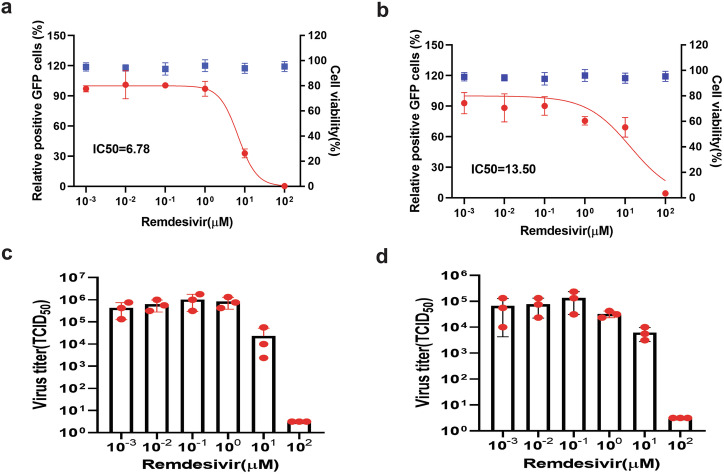
Evaluation of remdesivir using GFP reporter PRRSV-1 (a, c) and PRRSV-2 (b, d). The antiviral activity was assessed in MARC-145 cells using GFP-expressing reporter viruses LV-1’GFP-TRS6(GD) (PRRSV-1) and GD-7’GFP (PRRSV-2). Cells were infected at a MOI of 0.1 and treated for 24 h with varying concentrations of each compound. Left panels: Viral replication (red curves) was quantified by flow cytometric analysis of GFP-positive cells and normalized to untreated controls. Compound cytotoxicity (blue data points) was measured in uninfected MARC-145 cells using a colorimetric viability assay. Right panels: Viral titers in culture supernatants were determined 24 h post treatment. Data points represent mean ± SD from three independent experiments. Half-maximal inhibitory concentrations (IC_50_) were calculated using nonlinear regression analysis with a variable slope dose–response model in GraphPad Prism 8.0.

**Fig. 6:**
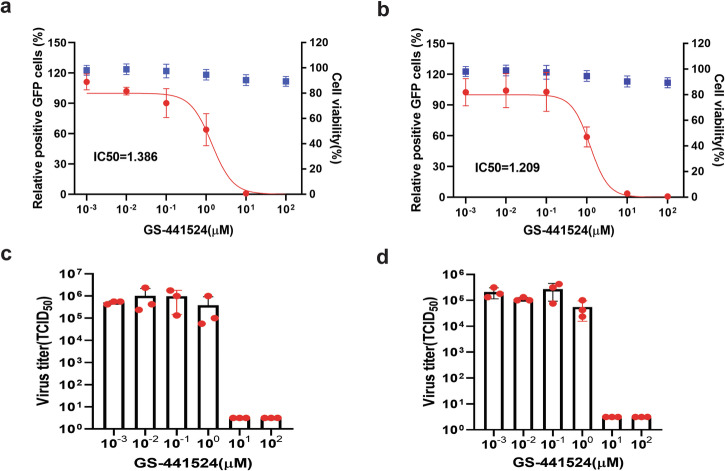
Evaluation of GS-441524 using GFP reporter PRRSV-1 (a, c) and PRRSV-2 (b, d). Upper panel: Viral replication (red curves) was quantified by flow cytometric analysis of GFP-positive cells and normalized to untreated controls. Blue data points: compound cytotoxicity. Lower panel: Viral titers in culture supernatants. Experimental conditions were identical as described in Fig. 5.

**Fig. 7:**
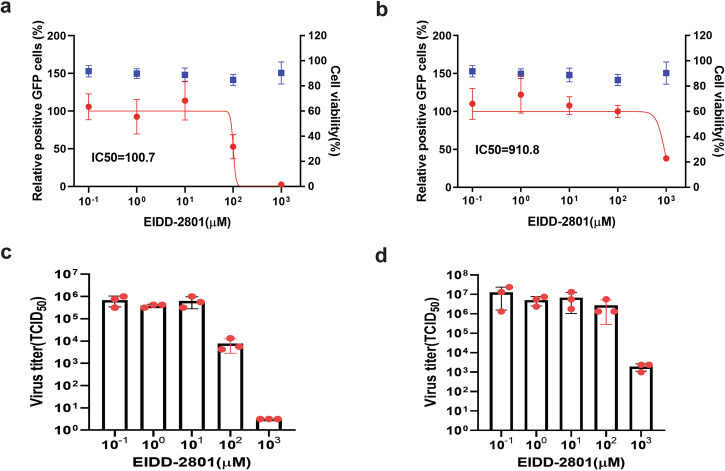
Evaluation of EIDD-2801 using GFP reporter PRRSV-1 (a, c) and PRRSV-2 (b, d). Upper panel: Viral replication (red curves) was quantified by flow cytometric analysis of GFP-positive cells and normalized to untreated controls. Blue data points: compound cytotoxicity. Lower panel: Viral titers in culture supernatants. Experimental conditions were identical as described in Fig. 5.

**Fig. 8:**
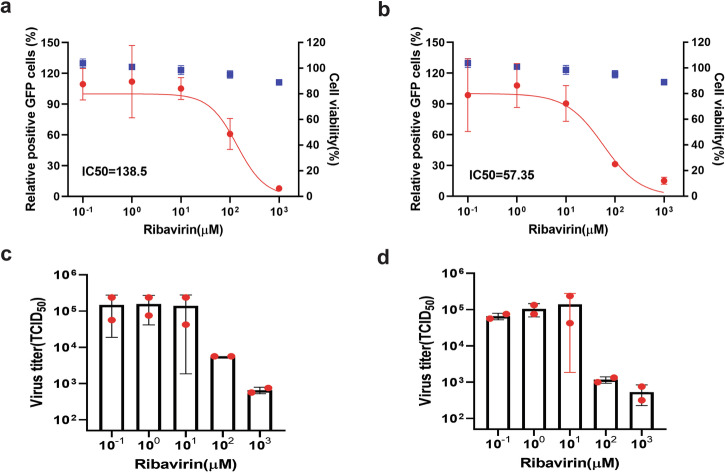
Evaluation of Ribavirin using GFP reporter PRRSV-1 (a, c) and PRRSV-2 (b, d). Upper panel: Viral replication (red curves) was quantified by flow cytometric analysis of GFP-positive cells and normalized to untreated controls. Blue data points: compound cytotoxicity. Lower panel: Viral titers in culture supernatants. Experimental conditions were identical as described in Fig. 5.

**Fig. 9:**
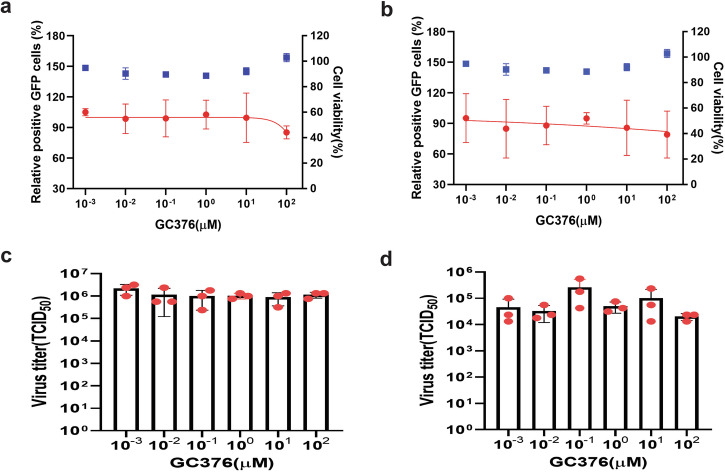
Evaluation of GC376 using GFP reporter PRRSV-1 (a, c) and PRRSV-2 (b, d). Upper panel: Viral replication (red curves) was quantified by flow cytometric analysis of GFP-positive cells and normalized to untreated controls. Blue data points: compound cytotoxicity. Lower panel: Viral titers in culture supernatants. Experimental conditions were identical as described in Fig. 5.

MARC-145 cells were infected with the GFP-expressing PRRSV-1 or PRRSV-2 at a MOI of 0.1. The infected cells were then incubated for 24 h in the presence of each compound, using a tenfold serial dilution ranging from 1 nM to 100 µM. To quantify the antiviral effects, we employed two complementary methods: (i) GFP-expressing cells were visualized by fluorescence microscopy (Supplementary Fig. 5) and their percentage was determined using flow cytometry, allowing for the calculation of half-maximal inhibitory concentration (IC_50_) values for each drug; (ii) virus titers in the cell culture supernatants were measured with the TCID_50_ assay to corroborate the flow cytometry results and provide a direct measure of viral replication inhibition. Prior to the inhibition assays, the cytotoxicity of each antiviral compound was assessed using colorimetric assay, and no adverse effects on cell viability were observed at the tested concentrations (Figs. 5–9, blue dots).

Antiviral activity assays revealed distinct efficacy profiles among the tested compounds against both PRRSV-1 and PRRSV-2. Remdesivir significantly inhibited replication of both PRRSV-1 and PRRSV-2 in MARC-145 cells at concentrations starting from 10 µM, with IC_50_ values of 6.78 µM and 13.50 µM, respectively. The observed reduction in GFP-positive infected cells correlated well with decreased virus titers in the culture supernatant in a concentration-dependent manner (Fig. 5).

GS-441524, the main plasma metabolite of remdesivir also showed potent inhibition of both PRRSV-1 and PRRSV-2 replication at concentrations starting from 10 µM, with lower IC_50_ values of 1.4 µM and 1.2 µM, respectively. This compound also significantly reduced viral titers in the culture medium at the effective concentration (Fig. 6).

EIDD-2801 (molnupiravir) showed inhibitory effects at higher concentrations, with 100 µM significantly reducing PRRSV-1 replication and 1000 µM required for PRRSV-2. The IC_50_ values for EIDD-2801 against PRRSV-1 and PRRSV-2 were 100.7 µM and 910.8 µM, respectively, with corresponding reductions in viral titers in the supernatant of treated cells (Fig. 7).

Ribavirin significantly inhibited replication of both PRRSV-1 and PRRSV-2 at 100 µM. The IC_50_ values for ribavirin were 138.5 µM and 57.4 µM against PRRSV-1 and PRRSV-2, respectively, with significant reductions in virus titers at these concentrations (Fig. 8).

Conversely, GC376, a broad-spectrum antiviral targeting the main protease (3CL^pro^) of coronaviruses, showed no inhibitory effect on PRRSV-1 or PRRSV-2 replication. Neither GFP-positive cells nor virus titers in the medium were affected by GC376 treatment, even at the highest tested concentration of 100 µM (Fig. 9).

In summary, all the tested compounds targeting the RdRp of coronaviruses, remdesivir, GS-441524, EIDD-2801, and ribavirin exhibited significant inhibitory effects on the replication of both PRRSV-1 and PRRSV-2. In contrast, the compound targeting the main protease of coronaviruses, GC376, showed no activity. These findings highlight the effectiveness of using GFP reporter PRRSV viruses for antiviral drug screening assays.

### Structural model of the PRRSV polymerase

We analyzed similarities between the RdRp structures of arteri- and coronaviruses to explain the observed inhibitory effects. Several structures of the RdRp (nsp12) of SARS-CoV-2 reflecting various stages of the replication process have been resolved^24,25,49,50^. Since no experimental structures of arterivirus RdRps are available, we used AlphaFold3 to predict the RdRp (nsp9) structures of PRRSV-1 and PRRSV-2 reference strains Lelystad and VR-2332, respectively. The models showed high quality based on the predicted local distance difference test (pLDDT) scores. Furthermore, both nsp9 structures are virtually identical, with a root mean square deviation (RMSD) of 0.377 Å, confirming their reliability (Supplementary Fig. 6).

**Fig. 10:**
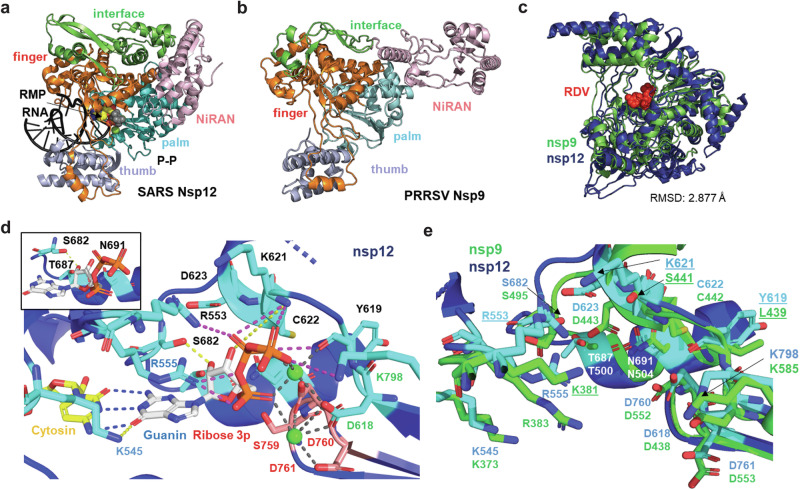
Structural comparison of the RNA-dependent RNA polymerases (RdRps) of SARS-CoV-2 (nsp12) and PRRSV (nsp9). Cartoon representations of SARS-CoV-2 nsp12 (**a**; experimentally determined structure, PDB: (7BV2) and PRRSV nsp9 (**b**; AlphaFold3-predicted structure). Domains are color-coded and labeled. **c** Structural alignment of SARS-CoV-2 nsp12 and PRRSV nsp9 RdRps. The root mean square deviation (RMSD) value reflects the degree of structural similarity. The NiRAN and interface domains were excluded from the alignment. RDV: remdesivir. **d** Catalytic center of SARS-CoV-2 nsp12 (PDB: 7UOB) bound to GTP. The catalytic motif S759-D760-D761 is highlighted in orange. Dashed lines indicate polar interactions: gray for Mg^2+^ coordination (green spheres), magenta for phosphate–nsp12, yellow for ribose/base–nsp12, and blue for guanine–cytosine base pairing. Inset: close-up of residues interacting with the GTP ribose. **e** Superposition of the SARS-CoV-2 nsp12 catalytic center (as shown in **d**) with the predicted PRRSV nsp9 structure. Catalytic residues in nsp9 are shown as green sticks; substitutions relative to SARS-CoV-2 are underlined. GTP is omitted for clarity.

The PRRSV nsp9 shares a similar domain organization with SARS-CoV-2 nsp12, comprising an N-terminal nucleotidyltransferase (NiRAN) domain, an interface region and the C-terminal polymerase domain, which is subdivided into the characteristic finger, palm and thumb subdomains (Fig. 10a, b). However, PRRSV nsp9 is substantially shorter (686 amino acids) compared to SARS-CoV-2 nsp12 (932 amino acids). The NiRAN domain in SARS-CoV-2 is associated with nsp9 N-terminus modification activities (NMPylation, RNAylation, and deRNAylation/capping); nsp9 itself assists in the replication and transcription of the SARS-CoV-2 genome. In contrast, only nucleotidyltransferase activity has been described for the Equine arteritis virus^51–53^. Both structures are different, only very poor alignment of a few amino acids is possible that do not encompass the GDP-binding site in nsp12 (Supplementary Fig. 6), indicating that their mechanism of action is different.

In contrast. the C-termini containing the core polymerase activity of nsp9 and nsp12 align remarkably well (RMSD score ~3 Å) despite low amino acid homology (15.0% identity, 24.5% similarity) (Fig. 10c). This allows to investigate whether the crucial amino acids involved in catalysis of nsp12 are conserved in nsp9. The SARS-CoV-2 RdRp active site comprises seven conserved catalytic motifs (A-G), with A-E in the palm subdomain and F-G in the finger subdomain. Entry routes for primer-template and exit routes for the nascent strand, are positively charged and solvent-accessible in nsp12. Comparing the electrostatic surface potential of nsp12 and nsp9 shows that the later also has a positively charged surface in the same region which might have the same purpose (Supplementary Fig. 6). Incoming nucleotides are recognized by K545 and R555 in the motif F of the finger domain, which interact, depending on the specific nucleotide, with the base and/or the α-phosphate. This induces a rotation of the RdRp motif A of the finger domain to close around the nucleoside phosphate (NTP) substrate. This (i) disrupts the polar D618–K798 interaction observed in the apo-RTC repositioning D618 and Y619 (motif A) to coordinate (together with D760 and D761, motif C) the two catalytic Mg^2+^ ions and K798 to interact with the NTP γ-phosphate, (ii) promotes the formation of a hydrogen-bonding network through D623 that enables binding of the substrate ribose 2’-OH by motif B residues S682, T687 and N691, (iii) enables H-bonding interactions between the β- and γ-phosphates and motif A residues K621 or C622. The incoming nucleotide forms a Watson-Crick base pair with the template nucleotide^24^ (Fig. 10d).

Structural alignment of nsp12 with nsp9 reveals mostly identical amino acids at crucial positions, with three exceptions: (i) Y619 in nsp12 aligns with L439 in nsp9, (ii) K621 in nsp12 with S441 in nsp9. Both are unlikely to have an impact on the catalytic reaction, since the main chain atoms coordinate the Mg^2+^ ion and interact with γ-phosphate, respectively and (iii) R553 in nsp12 aligns with a similar amino acid, K381 in nsp9 (Fig. 10e). Given the similar folding and presence of functionally equivalent amino acids at catalytically crucial positions, we speculate that the catalytic mechanism of nsp9 is likely very similar to that of nsp12.

**Fig. 11:**
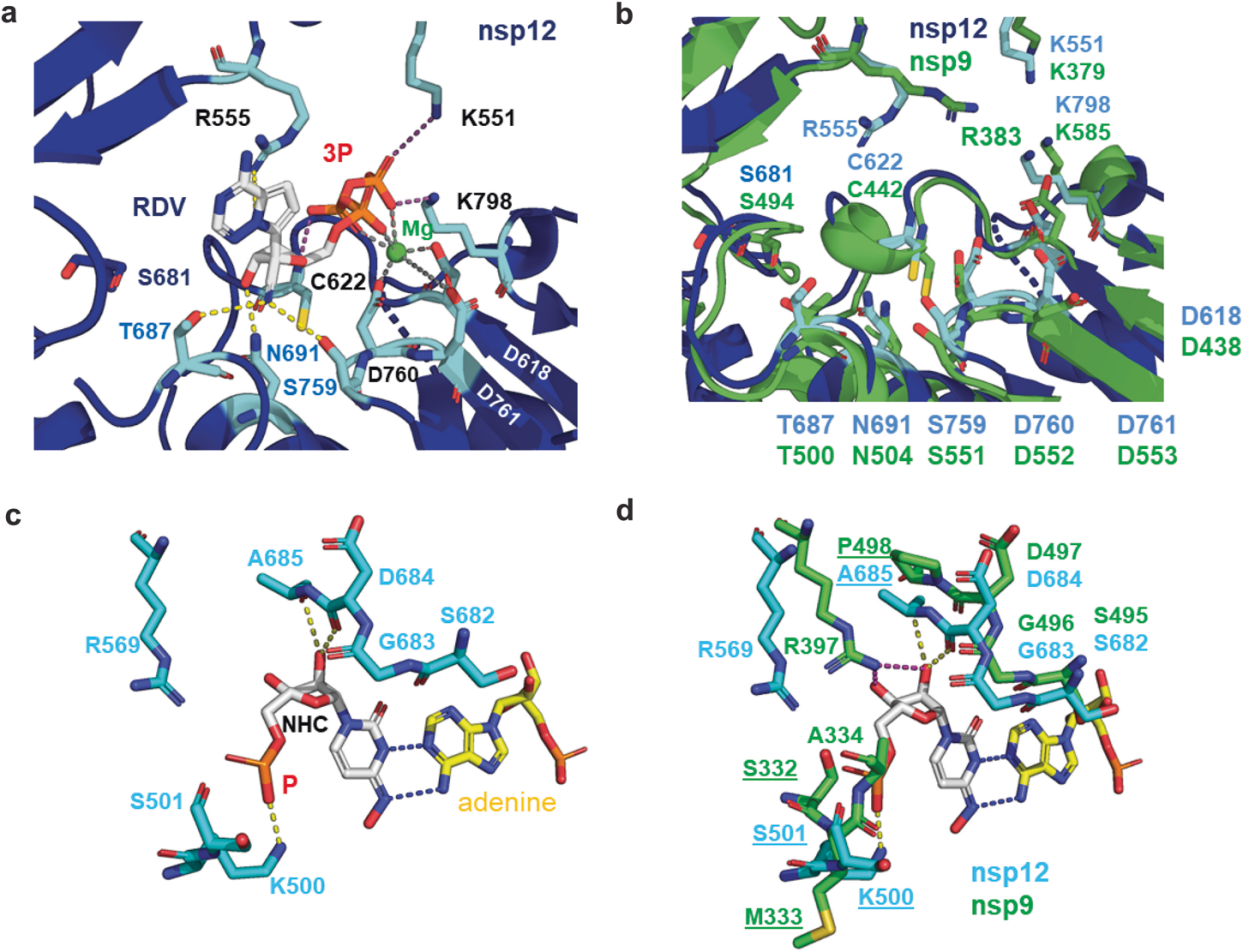
Structural comparison of SARS-CoV-2 nsp12 bound to remdesivir and molnupiravir with aligned residues in PRRSV nsp9. **a** Structural detail of the SARS-CoV-2 replication–transcription complex bound to remdesivir triphosphate in the pre-catalytic state (PDB: 7UO4). Remdesivir-interacting residues in nsp12 are shown as cyan sticks. Dashed lines indicate polar interactions: gray for Mg^2+^ coordination (green spheres), magenta for phosphate–nsp12 interactions, and yellow for ribose/base–nsp12 interactions. **b** Superposition of remdesivir-interacting residues in nsp12 (as shown in a) with the predicted structure of PRRSV nsp9. Aligned residues in nsp9 are shown as green sticks. **c** Structural detail of SARS-CoV-2 nsp12 bound to β-D-N4-hydroxycytidine (NHC), the active metabolite of molnupiravir, base-paired with adenine in the template strand (PDB: 7OZU). NHC is shown as white sticks; the phosphate group is highlighted in orange. Interacting residues in nsp12 are shown as cyan sticks. **d** Superposition of molnupiravir (NHC)-interacting residues in nsp12 (as shown in c) with the predicted structure of PRRSV nsp9. Aligned residues in nsp9 are displayed as green sticks.

We used the nsp12 structure bound to remdesivir to explain its inhibition of both SARS-CoV-2 and PRRSV replication^25^. Remdesivir and its main cellular metabolite GS-441524 are intracellularly phosphorylated to the active triphosphate form (RDV-TP), competing with ATP for incorporation into the growing viral RNA chain. Remdesivir’s 1’-cyano moiety provides 2-3-fold higher selectivity of RDV-TP over ATP by projecting into a hydrophilic pocket formed by T687, N691 (motif B), and S759 (motif C). This incorporation stalls RNA synthesis after three additional nucleotides due to a translocation barrier caused by the 1’-cyano-group and S681 in nsp12. Other crucial residues for RDV-TP binding include: (i) R555: interacts with the base, (ii) K551, C662, K798: interact with the phosphates, and (iii) D718, D760, D761: coordinate Mg^2+^^24,25,54^ (Fig. 11a). Structural alignment of nsp9 and nsp12 revealed identical residues at these crucial positions (including S681) in nsp9 (Fig. 11b), This structural conservation explains the comparable IC_50_/EC_50_ values observed for remdesivir and GS-441524 treatment in SARS-CoV-2^45^, PRRSV-1, and PRRSV-2 (see Table 2).

**Table 2:** Comparative summary of antiviral drug efficacy against SARS-CoV-2 and PRRSV

Antiviral	Drug class	Approved for	Inhibition of SARS-CoV-2 (µM)	Inhibition PRRSV-1 (IC_50_, µM)	Inhibition PRRSV-2 (IC_50_, µM)
Remdesivir (Verkluy)	Adenosine analogue	COVID-19	0.01 (EC_50,_ HAE) 0.28 (EC_50,_ Calu3) 1.65 (EC_50,_ Vero) (Pruijssers, 2020)	6,78	13,50
GS-441524 (active form of remdesivir)	Adenosine analogue	feline infectious peritonitis (off-label)	0.62 (EC_50,_ Calu3) 0.47 (EC_50,_ Vero) (Pruijssers, 2020)	1,39	1,21
Molnupiravir (Lagevrio)	Cytidine analogue	COVID-19 (under review)	0.30 (IC_50,_ Vero) 0.08 (IC_50,_ Calu3) (Sheahan, 2020)	100,7	910,8
Ribavirin (Rebetol)	Guanosine analogue	Hepatitis C	70 (IC_50,_ Caco) (Bojkova, 2020)	138,5	57,35
GC376	Protease inhibitor	Preclinical stage	0.92 (EC_50,_ Vero) (Vuong, 2020)	No	No

This table lists the antiviral compounds tested, their trade names, drug classes, and approved clinical indications. The table presents the published IC_50_ or EC_50_ values for SARS-CoV-2 in various cell lines (human airway epithelial cell cultures (HAE), Calu-3, Vero, and Caco-2) and the corresponding IC_50_ values for PRRSV-1 and PRRSV-2 in MARC-145 cells. References for the published SARS-CoV-2 data are provided in parentheses.

Molnupiravir also RdRp of SARS-CoV-2, but its inhibition mechanism is different. RdRp uses the active form of molnupiravir, ß-d-N4-hydroxycytidine (NHC) triphosphate, as a substrate instead of cytidine triphosphate or uridine triphosphate. When the resulting RNA is used as a template, NHC directs incorporation of either G or A, leading to mutated RNA products and hence to non-functional virus. Figure 11c shows SARS-CoV-2 nsp12 with NHC base-paired with adenine in the template strand. The following residues in nsp12 form hydrophilic interactions with NHC: (i) K500 with α-phosphate; (ii) main-chain atoms of A685 and D686 with the ribose. In nsp9 these residues are altered: K500 is replaced by M333, which does not interact with the α-phosphate, and A685 is replaced by P498, which causes some distorting of the local structure. In addition, the side chains of R569 and its homolog R397 are at a different position, such that the latter can from additional interactions with the ribose of NHC^55^ (Fig. 11d). These differences may partly explain why molnupiravir requires 1000 to 10,000 times higher concentrations to inhibit PRRSV-1 and PRRSV-2 replication compared to SARS-CoV-2 replication^46^ (Table 2).

In arteriviruses, nsp4 corresponds functionally to nsp5, the main protease of SARS-CoV-2^56,57^. Both belong to the 3C-like protease family with similar substrate specificities, recognizing cleavage sites with a conserved glutamine residue in the P1 position. However, no meaningful alignment of both experimentally determined structures is possible (Supplementary Fig. 7), explaining why the GC376 inhibitor targeted to 3CL^pro^ of SARS-CoV-2 had no effect on PRRSV replication.

## Discussion

In this study, we present a novel approach for the rapid construction of infectious clones of PRRSV using a yeast-based TAR cloning system. This method enables the assembly of overlapping DNA fragments covering the complete viral genome in yeast, resulting in a full-length viral cDNA (Fig. 1). The TAR cloning has previously been employed to create the first infectious clones for various coronaviruses^58,59^.

The TAR cloning system offers several significant advantages over traditional labor-intensive methods: (1) It eliminates the need for intermediate cloning steps, thereby reducing the risk of introducing errors and minimizing the need for subsequent correction. (2) The system enables efficient assembly of large viral genomes (up to several hundred kilobases) directly in yeast through homologous recombination^60^. (3) The assembled DNA is recovered from yeast as a circular molecule, facilitating subsequent manipulation in bacterial systems or direct transfection into mammalian cells. This ensures that viruses rescued from complete infectious cDNA clones exhibit minimal genetic variation. (4) TAR cloning simplifies mutagenesis and the insertion of foreign genes by incorporating modified DNA fragments during assembly. We demonstrated this by constructing six recombinant PRRSV clones expressing GFP using this approach. This system might be also useful to separate the genes of the structural proteins to manipulate them independently. (5) The entire process, from cDNA clone construction to virus rescue is extremely rapid, and can be completed within one week. These features highlight the TAR cloning system as a robust and versatile tool for generating infectious clones and performing precise genetic modifications of PRRSV and other arteriviruses.

Using TAR, we constructed and characterized GFP-expressing recombinant PRRSV-1 and PRRSV-2 strains, which provided insights into the interplay between foreign gene insertion sites, TRS, and genome stability. The insertion of the GFP cassette between ORF1 and ORF2 or between ORF7 and the 3**′**UTR yielded divergent outcomes in terms of recombinant virus recovery, GFP expression levels, and genetic stability, as summarized in Table 1 and illustrated in Figs. 2–4. Insertion between ORF1 and ORF2, which repurposes TRS2 to drive GFP expression while using TRS6 for Gp2 expression, proved more universally compatible across both PRRSV-1 and PRRSV-2. However, GFP expression levels were rather low (PRRSV-2) or the GFP gene was rapidly lost (PRRSV-1).

In contrast, insertion between ORF7 and the 3**′**UTR, utilizing TRS6 for GFP expression, exhibited virus species-specific differences. PRRSV-2 constructs demonstrated robust GFP expression and stability across eight serial passages. However, PRRSV-1 constructs required replacement of the native TRS6 including the flanking sequences with its PRRSV-2 counterpart to achieve virus recovery, but even then, GFP was rapidly lost during virus passage. The region between ORF7 and the 3**′**UTR is critical for genome replication and genome encapsidation. Therefore, inserting foreign genes in this area may create selective pressure favoring viral variants that excise non-essential genetic material through homologous recombination or replication errors. Variants that lost the GFP gene quickly outgrew the parental virus. The observation that PRRSV-2 tolerates 3**′**UTR-adjacent insertions better than PRRSV-1 may stem from species-specific differences in the architecture of cis-acting replication elements that facilitate template switching during discontinuous transcription^61^.

The strongest and most stable GFP expression was achieved by replacing PRRSV-1 TRS6 and its flanking nucleotides with those of PRRSV-2 and inserting the GFP cassette between ORF1 and ORF2 of the PRRSV-1 genome to drive Gp2 expression. The TRS sequences differ by only one nucleotide—TTAACC in PRRSV-2 versus TCAACC in PRRSV-1—but the flanking regions are shorter in PRRSV-2, resulting in a reduced distance to the Gp2 start codon.

Previous studies have demonstrated that the stability of heterologous gene expression, such as GFP, in arteriviruses and coronaviruses depends on the insertion site and transcription regulatory sequences (TRS). It is well-established that these effects are often difficult to predict and require empirical testing. For coronaviruses, stable expression (over 20 passages) is achieved by replacing non-essential genes, like 3a and 3b in transmissible gastroenteritis virus (TGEV) or the 3abc cluster in feline infectious peritonitis virus, while insertions in other regions (e.g., TGEV N/M genes) lead to rapid loss of expression^62,63^. Additionally, smaller inserted genes with lower protein toxicity enhance stability^64^.

For PRRSV, previous GFP-expressing constructs have shown varied stability depending on the insertion site. PRRSV-2 strain P129 maintained GFP expression for at least 37 passages when inserted between ORF1b and ORF2a^65^, whereas insertions in the nsp2 region of PRRSV-1 and PRRSV-2 and between ORF4 and ORF5a (PRRSV-2) were unstable within a few passages^29,31,66^.

In a prior study, GFP inserted between ORF1b and ORF2a in PRRSV-2 strain P129 showed stability over 37 passages^65^, whereas our most stable construct (PRRSV-1 with PRRSV-2 TRS6) lost expression by passage 19. Pei et al. used a synthetic TRS6 with optimized RNA secondary structure, which might enhance stability, indicating that precise TRS and flanking sequence design is critical for robust foreign gene expression.

The general propensity of positive RNA viruses to eliminate heterologous genes is largely due to high recombination frequencies, often driven by sequence duplication^67^. It might explain why PRRSV-2 TRS6 sequence in the PRRSV-1 Lelystad genome improved GFP stability compared to the native PRRSV-1 TRS6. While the core TRS sequences of PRRSV-1 and PRRSV-2 differ by only one nucleotide, a key difference lies in their flanking regions: the PRRSV-2 TRS6 has shorter flanking regions, leading to a reduced distance to the Gp2 start codon. Shorter homologous regions inherently decrease the likelihood of foreign gene excision through homologous recombination or replication errors.

The differential effect of insertion after gene 7 in PRRSV-1 LV or PRRSV-2 GD recombinant viruses might be explained by a RNA pseudoknot identified at the 3**′** end of arterivirus genomes, which is crucial for minus-strand RNA synthesis. It varies in base-pair composition between PRRSV-1 and PRRSV-2, potentially contributing to differential tolerance of foreign gene insertions in the ORF7/3**′**UTR region^61^.

Furthermore, mRNA abundance may affect viral replication; wild-type PRRSV sgmRNA-2 (encoding Gp2/E) is less abundant than sgmRNA-6^68^. Thus, repurposing TRS2 for GFP and using a TRS6 copy for Gp2/E might alter sgmRNA-2 transcriptional efficiency, potentially increasing Gp2 and E protein levels which might affect budding of infectious particles.

Our investigation using the GFP-expressing PPRSV-1 and PPRSV-2 demonstrates that polymerase-targeting antiviral compounds known to inhibit SARS-CoV-2—remdesivir, its main metabolite GS-441524, molnupiravir, and ribavirin—exhibit concentration-dependent inhibition of both PRRSV-1 and PRRSV-2 replication in MARC-145 cells, but with different efficacy (Figs. 5–9, summarized in Table 2).

To rationalize these effects, the 3D structure of PRRSV-1 and PRRSV-2 RdRp (nsp9) were predicted with AlphaFold. The high-quality models are identical to each other (RMSD: 0.38 Å) and very similar to RdRp (nsp12) of SARS-CoV-2 (RMSD: 2,88 Å), despite low amino acid homology (Supplementary Fig. 6). Especially the conservation of residues in the catalytic center between the PRRSV and SARS-CoV-2 RdRps suggests that the catalytic mechanism is very similar, reinforcing the paradigm that RdRp functional architecture is conserved across RNA viruses (Fig. 10). In contrast, the N-terminal NiRAN domain in nsp9 and nsp12 exhibit completely different folding (Supplementary Fig. 6).

The potent inhibition of PRRSV by remdesivir and its nucleoside precursor GS-441524, as evidenced by their IC_50_ values (6.78–13.5 μM for remdesivir; 1.21–1.39 μM for GS-441524), demonstrates a degree of efficacy that, while somewhat reduced, remains comparable to their activity against SARS-CoV-2 (EC_50_/IC_50_ values as low as 0.01–1.65 μM; Table 2). This similarity likely reflects a conserved mechanism of action: both compounds are metabolized to their active triphosphate forms, which compete with ATP for incorporation by the viral RdRp. Our structural alignment supports this, revealing that PRRSV nsp9 retains all key residues required for remdesivir-triphosphate binding (Fig. 11a, b), suggesting that the drug’s binding mode is preserved across these divergent viruses.

In contrast, molnupiravir exhibits markedly reduced potency against PRRSV (IC₅₀ = 100.7–910.8 μM) compared to SARS-CoV-2 (IC_50_ = 0.08–0.3 μM). This difference may be attributed to structural divergence in the RdRp active site, particularly in regions that interact with the active metabolite NHC-TP (Fig. 11c, d). Additionally, variations in how NHC-TP competes with CTP and UTP for incorporation into viral RNA may further diminish its efficacy in PRRSV.

Ribavirin, a broad-spectrum antiviral, displays similar IC_50_ values against both PRRSV (57.35–138.5 μM) and SARS-CoV-2 (~70 μM). This consistency aligns with ribavirin’s multiple mechanisms of action—including induction of lethal mutagenesis, inhibition of inosine monophosphate dehydrogenase, and immunomodulatory effects—which do not solely depend on direct incorporation by the viral RdRp. Such multimodal activity likely underpins its conserved efficacy across diverse RNA viruses^69^.

In contrast, the main protease inhibitor GC376, which is effective against SARS-CoV-2 (EC₅₀ = 0.92 μM), shows no inhibitory activity against PRRSV. While both the coronavirus main protease and PRRSV nsp4 are 3C-like serine proteases with similar substrate specificities^56,57^, fundamental structural differences between these enzymes (Supplementary Fig. 7) likely account for the lack of cross-inhibition.

Note, however, that comparison of IC_50_ and EC_50_ values between SARS-CoV-2 and PRRSV has several limitations. Published experiments with SARS-CoV-2 were mostly performed in Vero and Calu cells, whereas we investigated PRRSV inhibition in MARC-145, cells which may yield different results compared to primary PAMs, the natural target cells of PRRSV in pigs. Differences in IC_50_ values may therefore also reflect cell-specific differences in prodrug activation or off-target effects. Comparison of IC_50_ values also assumes identical drug binding kinetics to the polymerase, but differences in nsp9/nsp12 processivity could influence drug efficacy. Furthermore, IC_50_ values depend on the exact assay conditions, such as MOI, timing of drug addition, and endpoint measurement. Additionally, the structural model of nsp9 relies on AlphaFold predictions, which may mispredict side-chain conformations. However, these uncertainties are unlikely to affect the main conclusions of this study.

This study establishes a yeast-based TAR cloning system as a robust platform for rapid assembly of stable PRRSV infectious clones, facilitating precise genetic engineering and enabling high-throughput antiviral screening. Key findings reveal that polymerase-targeting antivirals, effective against SARS-CoV-2, exhibit comparable or moderately reduced efficacy against PRRSV, while SARS-CoV-2 main protease inhibitors show no activity. Structural analysis provides mechanistic insight: the high similarity between the experimentally resolved SARS-CoV-2 polymerase and the AlphaFold-predicted PRRSV polymerase explains conserved drug susceptibility, whereas divergent protease architectures account for the lack of cross-reactivity. The species-specific instability of GFP-expressing PRRSV constructs highlights the critical need to optimize insertion sites and TRS for developing reliable viral vectors. These advances position the TAR system as a transformative tool for accelerating research on arteriviruses, with direct implications for next-generation vaccines.

## Methods

### Cells, virus strains

HEK 293T (human embryonic kidney) and MARC-145 (simian kidney epithelial) cells were maintained as adherent cultures in Dulbecco’s Modified Eagle’s Medium (DMEM) supplemented with 10% fetal calf serum (FCS), 100 IU/mL penicillin, and 100 μg/mL streptomycin at 37 °C in a humidified atmosphere containing 5% CO_2_. PAMs were isolated from 10- to 11-week-old pigs by a lung lavage as described previously^70^. PAMs were maintained in RPMI medium (PAN, Aidenbach, Germany) containing 10% fetal calf serum (FCS) (Perbio, Bonn, Germany), 100U of penicillin per ml, and 100 mg of streptomycin per ml at 37 °C in an atmosphere with 5% CO_2_ and 95% humidity. The animal work was approved by the governmental agency, the Landesamt für Gesundheit und Soziales (LAGeSo) in Berlin, Germany (approval number T0297/17).

The PRRSV-2 virus, strain XH-GD (Genbank accession: EU624117.1) is a highly pathogenic strain which was first isolated in Xinhui, a district of Jiangmen city in Guangdong province, China. This strain served as the primary virus for the study and was rescued from the infectious cDNA clone pPRRSV-WT, generously provided by Prof. Guihong Zhang (South China Agricultural University). The Lelystad virus (LV), a low-pathogenicity PRRSV-1 prototype strain adapted for growth in MARC-145 cells (Genbank accession: M96262.2) was kindly provided by Prof. Hans Nauwynck (Ghent University, Belgium).

### Yeast and bacterial strains

The highly transformable *S. cerevisiae* strain VL6-48N (MATα, his3-Δ200, trp1-Δ1, ura3-Δ1, lys2, ade2-101, met14, cir°)^59^, provided by Prof. Volker Thiel (University of Bern, Switzerland) was used for TAR cloning. Yeast cells were initially cultured in YPD broth, and transformed cells were selected on synthetic-defined (SD) agar plates lacking histidine (SD-His). *E. coli* 10G BAC-optimized electrocompetent cells (LGC Biosearch Technologies) were used to propagate the TAR cloning vector pBAC-His3.

### Assembly of full-length cDNA clone of PRRSV-1 and PRRSV-2 by TAR cloning

To assemble a viral genome using TAR cloning, the first step is to amplify DNA fragments covering the complete genome of PRRSV. The adjacent DNA fragments were designed to overlap by at least 50 nucleotides. To clone the Lelystad strain of PRRSV-1, RNA was isolated from infected MARC-145 cells using TRIzol reagent (Thermo Fisher Scientific), reverse transcribed into cDNA with SuperScript IV reverse transcriptase (Thermo Fisher Scientific), and then amplified as overlapping fragments using the primers listed in Supplementary Table 1. Similarly, a plasmid containing a full-length cDNA clone of the XH-GD strain of PRRSV-2 (pPRRSV-WT) was used as a template to generate overlapping fragments. Since we synthesized cDNA from viral RNA stock for PRRSV-1 we used five ~3-kb fragments to minimize PCR amplification errors, ensuring high-fidelity assembly via TAR cloning. For PRRSV-2, an existing infectious cDNA clone enabled assembly with two larger fragments, leveraging its complete genomic sequence. PCR amplification was done with high-fidelity PrimeSTAR GXL DNA polymerase (Takara), following the manufacturer’s instructions.

Yeast transformation was performed using the lithium acetate (LiAc)/DNA/PEG method, as described by Thao in 2020^39^. Briefly, *S. cerevisiae* strain VL6-48N was cultured overnight in YPD broth at 30 °C with shaking at 200 rpm. The following day, the culture was diluted 1:5 in pre-warmed YPD broth and incubated at 30 °C until it reached OD_600_ of 1. For each transformation, 3 mL of culture was harvested by centrifugation (2500 × *g*, 22 °C, 5 min), washed once with the LiAc buffer (0.1 M LiAc, 10 mM Tris-HCl, 1 mM EDTA, pH 7.5), resuspended in 1 mL of the same LiAc buffer and incubated at 30 °C for 1 h.

After incubation cells were pelleted (2500 × *g*, 22 °C, 5 min) and resuspended in 50 μL of LiAc buffer. A mixture of denatured salmon sperm DNA and all overlapping DNA fragments (including the TAR vector, 500 ng each) resuspended in no more than 55 μL was added to the cells. Next, 500 μL of 40% polyethylene glycol (PEG 3350) and 67 ul of 10% dimethyl sulfoxide (DMSO) were added to the DNA/cell mixture. The mixture was incubated at 30 °C without agitation for 30 min. The mixture was then heat-shocked at 42 °C for 25 min in a water bath, and transformed cells were resuspended in 1 mL of YPD medium and incubated at 30 °C for 1 h with agitation at 200 rpm. Finally, the transformed yeast cells were plated on SD-His plates and incubated at 30 °C for 2 days until colonies appeared.

Subsequently, selected yeast colonies were suspended in 5 mL of SD-His liquid medium and incubated overnight at 30 °C with shaking at 200 rpm. The entire culture was then used for plasmid extraction to isolate pBAC-His3 carrying the complete genome of PRRSV. Plasmid extraction was performed using the QIAGEN Miniprep Plasmid Kit, with protocol modifications to enable efficient lysis of yeast cells. Specifically, the resuspension buffer P1 (50 mM Tris-Cl, 10 mM EDTA, 100 µg/mL RNase A, pH 8.0) was supplemented with zymolyase solution (1:10) and β-mercaptoethanol (1:100) to facilitate yeast cell wall digestion.

The purified recombinant plasmids were subsequently transformed into E. cloni 10G BAC-optimized electrocompetent cells (LGC Biosearch Technologies) and plated on LB agar plate containing chloramphenicol (34 μg/mL). DNA was extracted from bacterial clones using a standard miniprep isolation. To overcome the typically low yield associated with 5 mL cultures (~100–200 µg/µL), we scaled up the culture volume to 20–30 mL and employed multiple flow-through steps. The resulting plasmid preparations exhibited sufficient purity and concentration for direct transfection into mammalian cells. Assembly of the viral genome was next assessed by restriction fragment length polymorphism (RFLP) analysis, and sequence accuracy was verified by whole-genome nanopore sequencing (Eurofins Genomics).

### Construction of cDNA clones of PRRSV containing an expression cassette of GFP gene

The infectious cDNA of PRRSV was developed as a vector to express green fluorescent protein (GFP) through an additional subgenomic RNA. The expression cassette of GFP gene was inserted into the PRRSV genome at two sites, ORF1/ORF2 and ORF7/3**′**UTR, respectively. In constructs pGD-1’GFP of PRRSV-2 and pLV-1’GFP of PRRSV-1, the GFP gene fused with a TRS6 sequence and flanking from corresponding strain at the 3**′** end, was inserted between ORF1 and ORF2 of genome. These sequences are: TGGTTCCGCGGCAACCCCTTTAACCAGAGTTTCAGCAGAACA in XH-GD strain, GTCCTCGAAGGGGTTAAAGCTCAACCCTTGACGAGGACTTCGGCTGAGCA in Lelystad virus strain. In constructs pGD-7’GFP of PRRSV-2 and pLV-7’GFP of PRRSV-1, the GFP gene fused with a TRS6 sequence and flanking at 5**′** end was inserted between ORF7 and 3**′**UTR of genome. In another two constructs of PRRSV-1 pLV-1’GFP^TRS(GD)^ and pLV-7’GFP^TRS(GD)^, the original TRS6 sequence and flanking were replaced by its counterpart from XH-GD strain of PRRSV-2, was inserted at sites ORF1/ORF2 and ORF7/3**′**UTR in the genome of PRRSV-1, respectively. All these six cDNA infectious clones with expression of GFP gene were assembled through TAR cloning strategy in the yeast, as described for their wildtype. All DNA fragments used for assembly, except one containing expression cassette of GFP was synthesized, were amplified via PCR from template plasmids pBAC-His3 harboring the complete genome of PRRSV-2 pXH-GD or PRRSV-1 pLelystad virus. The integrity and accuracy of resulting plasmids were confirmed using restriction fragment length polymorphism (RFLP) analysis and whole-genome sequencing (Eurofins Genomics).

### Recovery of viruses

The plasmids (2.5 μg) containing the complete cDNA clones from reconstructed PRRSV were transfected into 80% confluent HEK 293T cells grown in 6-well plates using Lipofectamine 3000 (Thermo Fisher Scientific) as described by the manufacturer. Seventy-two hours after transfection cell culture media (P0 virus) were collected, cleared by low-speed centrifugation (5000 × *g*, 5 min), and 500 μl was used to infect MARC-145 cells grown to 80% confluency on 6-well plates. After incubation for 1 h at 37 °C, the inoculum was removed, cells were washed once with PBS, and further incubated in culture medium (DMEM with 2% FCS) for 72 h. Cells were then subjected to immunofluorescence assay using monoclonal antibody against the nucleocapsid (N) protein of PRRSV-2 or PRRSV-1. The supernatant collected from infected MARC-145 cells was defined as P1 virus.

### Virus growth kinetics

Sub-confluent MARC-145 cells in 24-well plates were infected with wildtype and reconstructed viruses from passage 1 (P1) at a MOI of 0.01 or 0.1. After 1 h incubation at 37 °C, cells were washed three times with PBS and incubated at 37 °C in 0.5 mL DMEM containing 2% FCS in a CO_2_ incubator. At certain time points (12, 24, 48, 72 and 96 h) post-infection, supernatants were collected and frozen at –80 °C until use. The viral titers were determined in MARC-145 cells with the endpoint assay 50% tissue culture infection dose (TCID_50_). The growth curve of the virus was generated using GraphPad Prism 8.

### SDS-PAGE and Western blotting

Proteins were separated by sodium dodecyl sulfate-polyacrylamide gel electrophoresis (SDS-PAGE) using 12% polyacrylamide gels and transferred onto polyvinylidene difluoride (PVDF) membranes (GE Healthcare). Membranes were blocked for 1 h at room temperature in a blocking solution (PBS containing 0.1% Tween-20 [PBST] and 5% skim milk powder). Subsequently, membranes were incubated overnight at 4 °C with primary antibodies diluted in blocking solution: a monoclonal antibody against the N protein PRRSV-1 (13E2, kindly provided by Prof. Hans Nauwynck, Ghent University, 1:1000), or a monoclonal antibody against the N protein of PRRSV-2 (DMAB28442, Creative Diagnostics, 1:3000). The same membranes were subsequently re-probed with a polyclonal anti-GFP antibody (16286-1-AP, Proteintech, 1:3000). For protein detection, membranes were washed three times with PBST for 10 min and incubated for 1 h at room temperature with the appropriate horseradish peroxidase (HRP)-conjugated secondary antibody: anti-mouse IgG (1706516, Bio-Rad Laboratories, 1:2000) or anti-rabbit IgG (Ab191866, Abcam, 1:5000). Chemiluminescent signals were developed using ECLplus reagent (Thermo Fisher Scientific) and visualized with a Fusion SL imaging system (Peqlab).

### Indirect immunofluorescence

Infected MARC-145 cells grown in 6-well plates were washed with PBS, fixed with 4% formaldehyde in PBS for 15 min at room temperature, and permeabilized with 0.2% Triton X-100 in distilled water for 7 min at room temperature. After blocking with 3% bovine serum albumin (BSA) in PBST for 30 min, cells were incubated with monoclonal antibody against N of PRRSV-2 (1:1000 dilution) or antibody against N of PRRSV-1 (1:200 dilution) for 1 h at room temperature. Cells were then washed with PBS and incubated with Alexa Fluor 488-conjugated anti-mouse IgG secondary antibody (1:1000 dilution). Images were acquired using a Axio Vert.A1 inverse epifluorescence microscope (Carl Zeiss).

### In vitro cytotoxicity assay

The cytotoxicity of GC376, molnupiravir, remdesivir, GS-441524, and ribavirin to MARC-145 cells was determined using the Cell Counting Kit 8 (Hycultec). Stock solutions of the drugs were prepared in 100% DMSO. MARC-145 cells were seeded in 96-well tissue culture plates and incubated at 37 °C for 24 h. Compounds were added at the indicated concentrations in DMEM medium (2% FCS), with three replicates per concentration. After 24 h, the compounds were removed, the cells were washed with PBS, and incubated with diluted colorimetric reagent for 1 h. The number of living cells was determined by measuring the absorbance at 450 nm with a microplate reader. Cell viability was calculated as a percentage relative to untreated controls.

### Antiviral assay

The antiviral efficacy of GC376, molnupiravir (EIDD-2801), remdesivir, its main metabolite GS-441524, and ribavirin against PRRSV-1 and PRRSV-2 replication was evaluated using flow cytometry and viral titer analysis. MARC-145 cells were seeded into 24-well plates with culture medium supplemented with 10% FCS and incubated for 24 h. The medium was removed, and cells were washed with PBS. Cells were infected with passage 3 recombinant GFP reporter PRRSV-1 and PRRSV-2 at a MOI of 0.1, with gentle shaking every 15 min to facilitate virus adsorption. After 1 h, the inoculum was removed, and the cells were washed with PBS. Fresh medium containing 2% FCS and varying concentrations of the antiviral drugs was added, and the plates were incubated at 37 °C with 5% CO_2_ for 24 h. Supernatants were collected and titrated using the TCID_50_ assay. Cells were detached with EDTA-trypsin, resuspended in PBS, and analyzed for GFP fluorescence using a CytoFlex flow cytometer (Beckman Coulter). The percentage of GFP-positive cells in drug-treated wells was normalized to untreated controls to calculate the relative fluorescence. The half-maximal inhibitory concentration (IC_50_) was calculated using nonlinear regression analysis and the dose-response (variable slope) equation in GraphPad Prism 8.0 software (Dotmatics).

### Prediction of the structure of nsp9 using AlphaFold 3

AlphaFold 3 model (Google DeepMind; https://alphafoldserver.com/) was used to predict the structures of nsp9 of the reference strains of PRRSV-1 (Lelystad virus) and PRRSV-2 (VR-2332) using the respective amino acid sequences as input. AlphaFold generates three confidence scores: (1) pTM score assesses the accuracy of the overall structure of the prediction at a 0–100 scale, where higher values indicate higher confidence. (2) pLDDT score: Indicates confidence in local structure prediction (0–100 scale). 90: Very high accuracy, 70–90: High accuracy, 50–70: Lower accuracy, <50: Potentially intrinsically unstructured region. The pLDDT score is saved in the B-factors field of the mmCIF file that contains a predicted structure. High-confidence areas (high B-factors) are red, while low-confidence areas (low B-factors) are blue. (3) Predicted Aligned Error (PAE) score: Calculated error of predicted distance for each residue pair. The figures were generated with PyMol (Schrödinger, LLC; https://pymol.org/2/).

## Supplementary information

Supplementary Information

## Data Availability

No datasets were generated or analysed during the current study.

## Code availability

There is no custom code associated with this submission.
