# Supplementary material for: Development of GFP-expressing infectious clones for PRRSV using TAR cloning for antiviral drug screening
Source: Npj Viruses. 2025 Sep 5;3:66. doi: 10.1038/s44298-025-00148-3 (PMC12413465; doi:10.1038/s44298-025-00148-3)
Supplement: Supplementary file 1 — Supplementary Information [file 44298_2025_148_MOESM1_ESM.pdf]

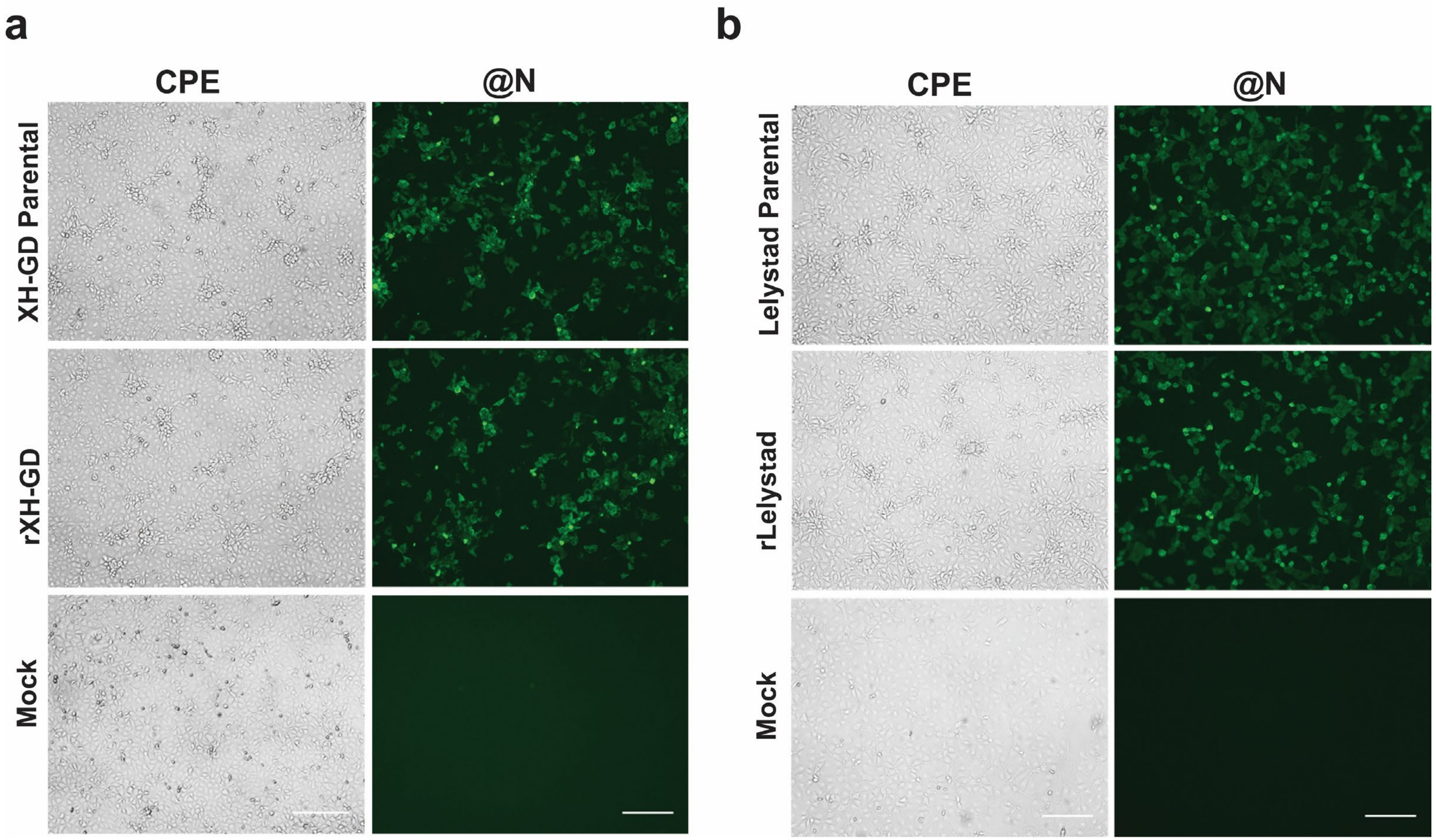

Supplementary Figure 1. Validation of virus rescue. a, b DNA from infectious clones pBAC-His3-XH-GD (PRRSV-2, a) and pBAC-His3-Lelystad (PRRSV-1, b) was isolated form *E. coli* and transfected into HEK 293T cells. After 72 hours, cell culture media were collected and typically 30% was used to infect MARC-145 cells. Infected cells developed characteristic cytopathic effects (CPE) 48–72 hours post-infection. Virus replication was further confirmed by immunofluorescent detection of the viral nucleoprotein (N). Mock-infected cells served as a negative control. Scale bar: 100 µm.

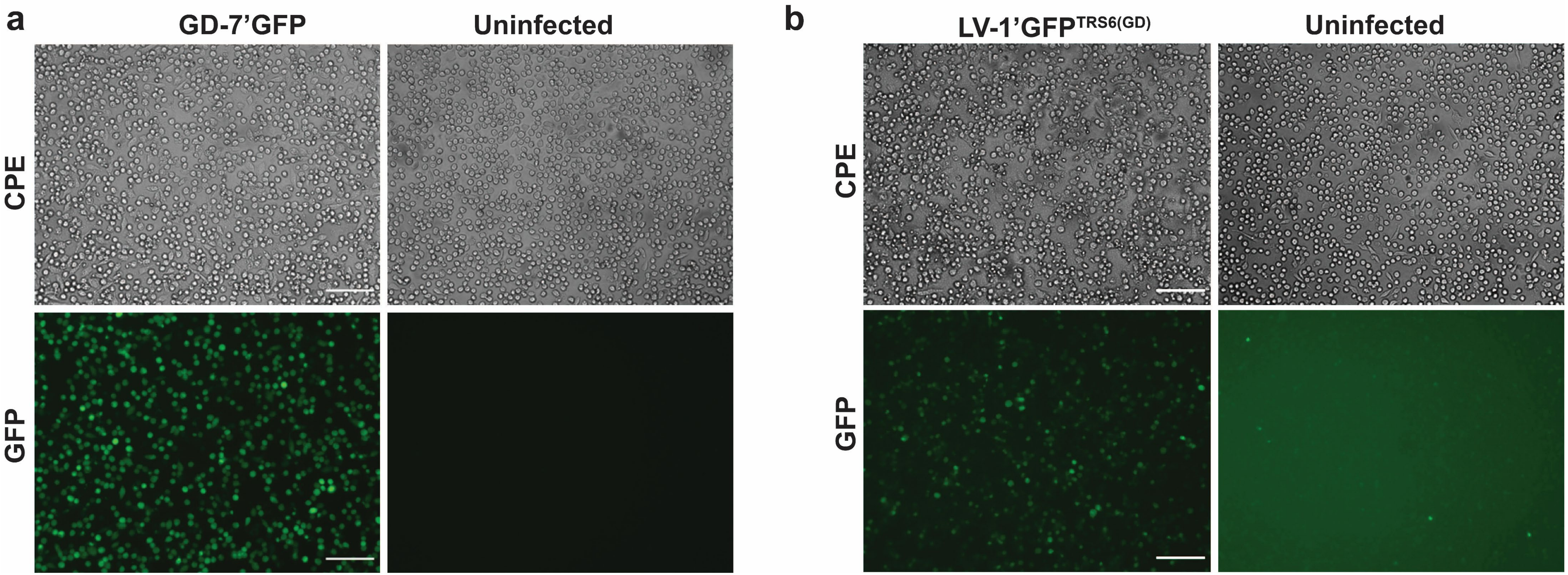

Supplementary Figure 2. GFP Expression in Porcine Alveolar Macrophages (PAMs) Infected with PRRSV Constructs. PAMs were infected at an MOI of 1 with passage 3 PRRSV-2 GD-7'GFP (a) or PRRSV-1 LV-1'GFP$^{TRS6(GD)}$ (b). At 24 hours post-infection, cells were visualized by bright-field and fluorescence microscopy to detect GFP expression. Scale bar, 100 µm

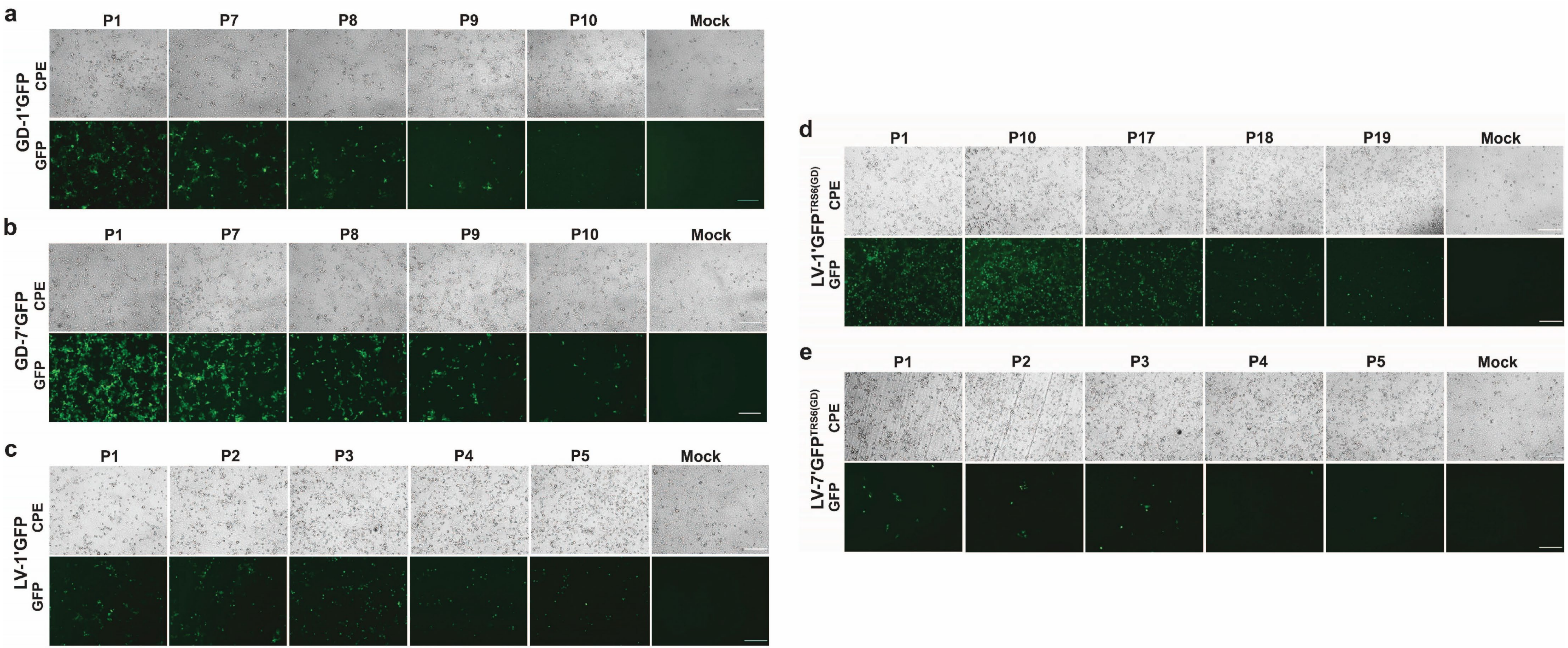

Supplementary Figure 3. Stability of GFP expression in PRRSV-1 and PRRSV-2 reporter viruses during serial passaging. To evaluate the stability of GFP expression, GFP-expressing PRRSV-1 and PRRSV-2 reporter viruses were serially passaged in MARC-145 cells. The number of passages (P) varied among the reporter viruses, depending on the stability of GFP expression. At each passage, infected MARC-145 cells were examined for cytopathic effects (CPE) and GFP fluorescence. Mock: uninfected MARC-145 cells. Pictures were captured at 20× magnification, Scale bar: 100 µm.

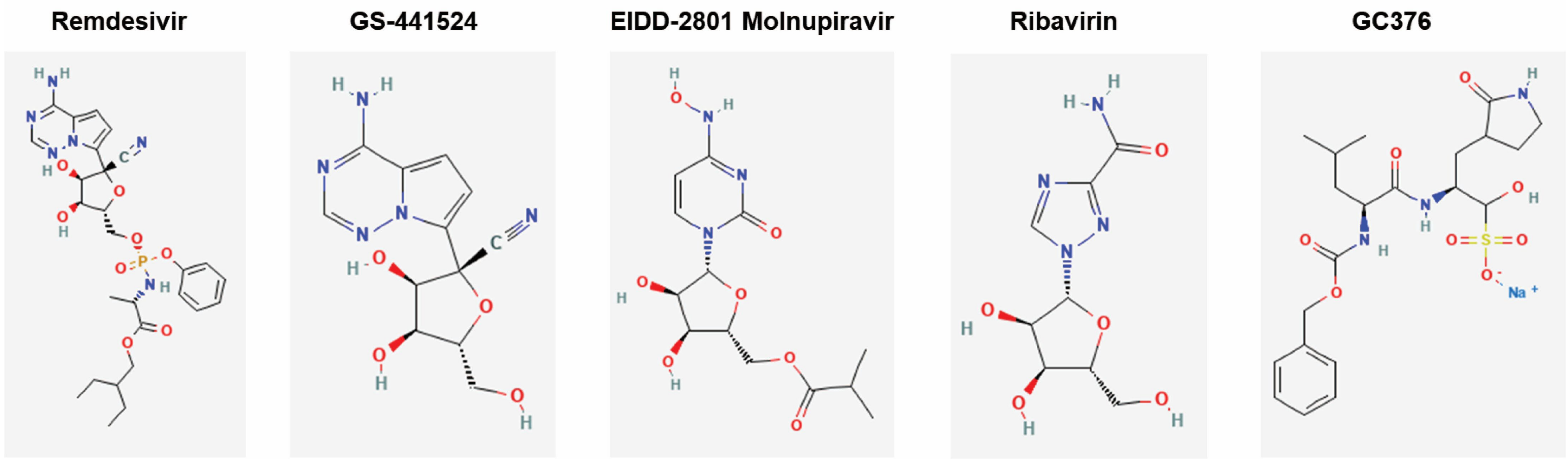

Supplementary Figure 4. Structural formulas of the antiviral compounds used in this study. The antivirals included nucleoside analogues and a viral protease inhibitor. Remdesivir, GS-441524, molnupiravir, and ribavirin are nucleoside analogues that inhibit viral replication by targeting the viral RNA-dependent RNA polymerase (RdRp). Remdesivir is a prodrug of an adenosine analog that is metabolized to its active triphosphate form and incorporated into viral RNA, leading to premature termination of RNA synthesis. GS-441524 is the main plasma metabolite of remdesivir and acts through the same mechanism. Molnupiravir (EIDD-2801) is a prodrug of β-D-N4-hydroxycytidine, which is incorporated into viral RNA in place of cytidine or uridine, introducing random mutations that accumulate during replication and ultimately result in lethal mutagenesis. Ribavirin is a guanosine analog with a multifaceted mode of action, including depletion of intracellular GTP pools, inhibition of viral RNA synthesis, and induction of error-prone replication, which contributes to its antiviral activity. GC376 is a protease inhibitor that blocks 3CL^{pro}, a protease common to many (+)ssRNA viruses, thereby preventing the viral polyprotein from maturing into its functional components. GC376 has demonstrated broad-spectrum activity against a range of coronaviruses, including SARS-CoV-2. Chemical structures were obtained from PubChem (https://pubchem.ncbi.nlm.nih.gov/).

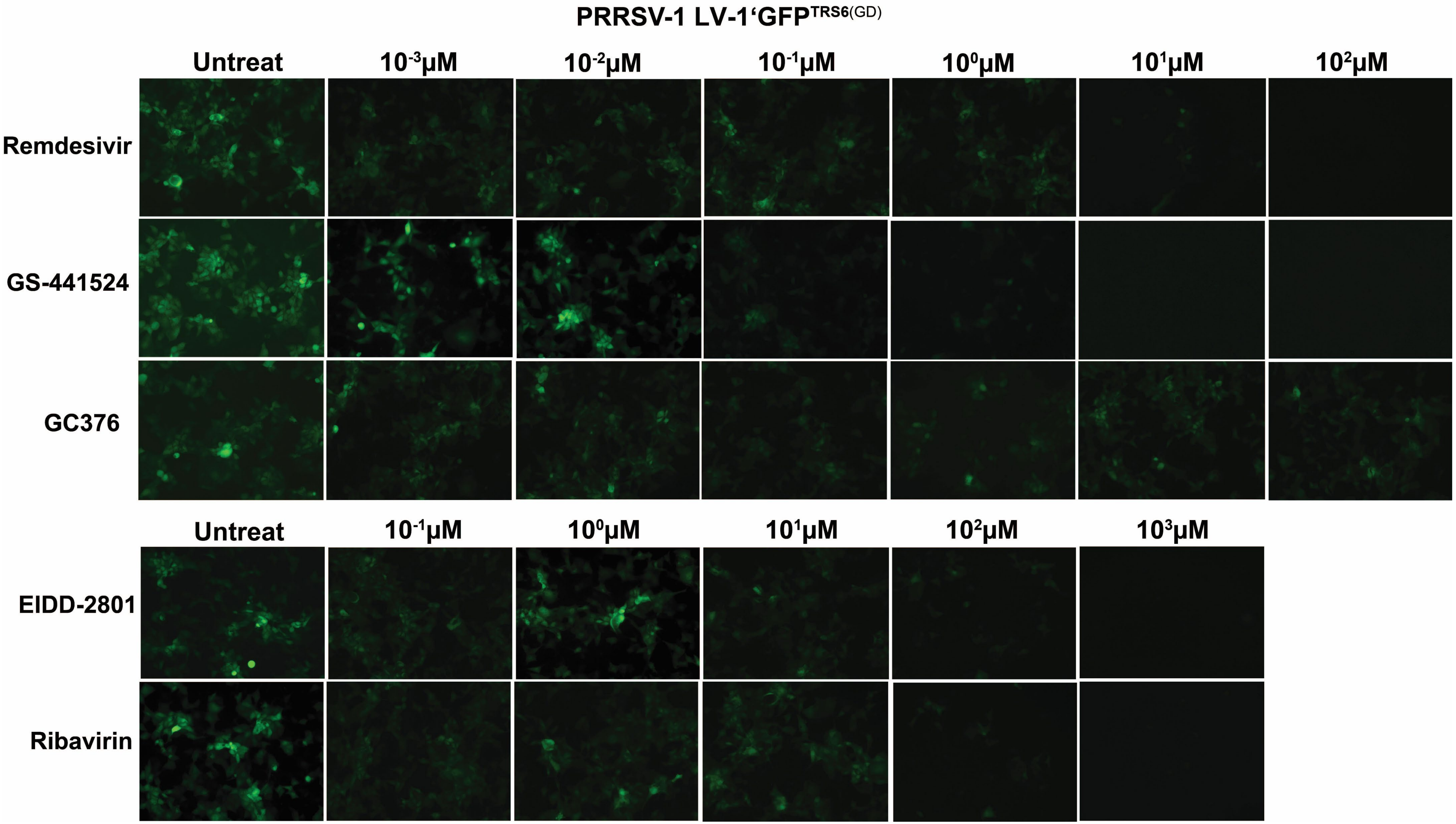

Supplementary Figure 5. Evaluation of antiviral compounds using GFP reporter PRRSV-1. MARC-145 cells were infected in triplicate with GFP reporter LV-1’GFP-TRS6(GD) at a MOI of 0.1, in the presence of increasing concentrations of the indicated antiviral compounds. After 24 hours GFP fluorescence was visualized to assess efficacy of each compound in inhibiting viral replication. Pictures were captured at 40× magnification. Slight variations in GFP expression within Fig. S5’s untreated panels reflect differences in CPE progression due to PRRSV infection.

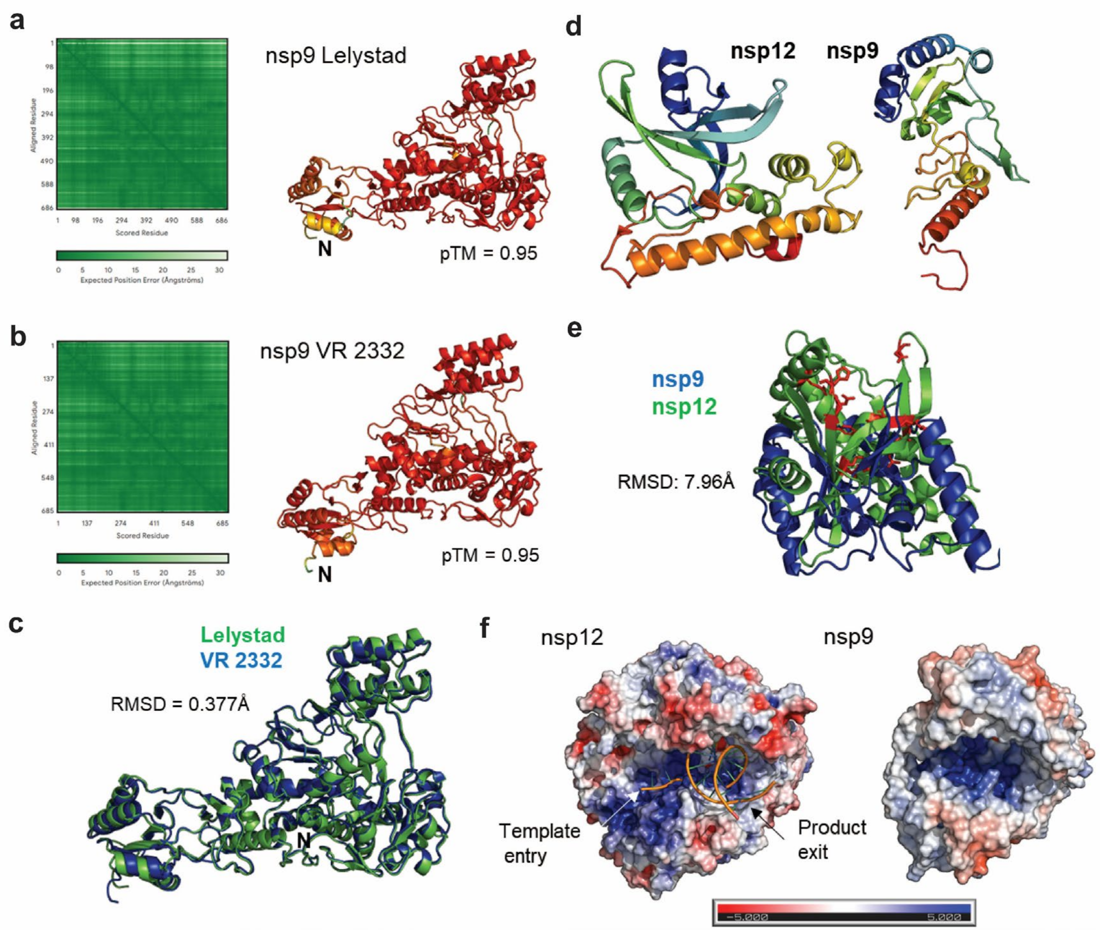

Supplementary Figure 6. AlphaFold models of nsp9 from PRRSV-1 and PRRSV-2. a, b Structural predictions of nsp9 from the PRRSV-1 prototype strain Lelystad (a) and the PRRSV-2 prototype strain VR-2332 (b), generated using AlphaFold3. The green matrix represents the Predicted Aligned Error (PAE) score, indicating the estimated positional error between pairs of residues. The red cartoon models display the predicted Local Distance Difference Test (pLDDT) scores, with rainbow coloring from blue (high confidence) to red (low confidence). The Predicted Template Modeling (pTM) score reflects the overall structural accuracy; values >0.8 indicate high-confidence models. **c** Structural alignment of nsp9 from PRRSV-1 and PRRSV-2. RMSD: root mean square deviation of the alignment. **d** Structural comparison of the N-terminal NiRAN domains of nsp12 and nsp9, color-coded from blue (N-terminus) to red (C-terminus). **e** Structural alignment of the N-terminal NiRAN domains of nsp9 and nsp12. Residues in nsp12 involved in GTP binding are shown as red sticks. **f** Surface electrostatic potential maps of nsp12 and nsp9, generated using the APBS electrostatics plugin in PyMOL. The scale bar indicates electrostatic potential ranging from –5 (red) to +5 (blue).

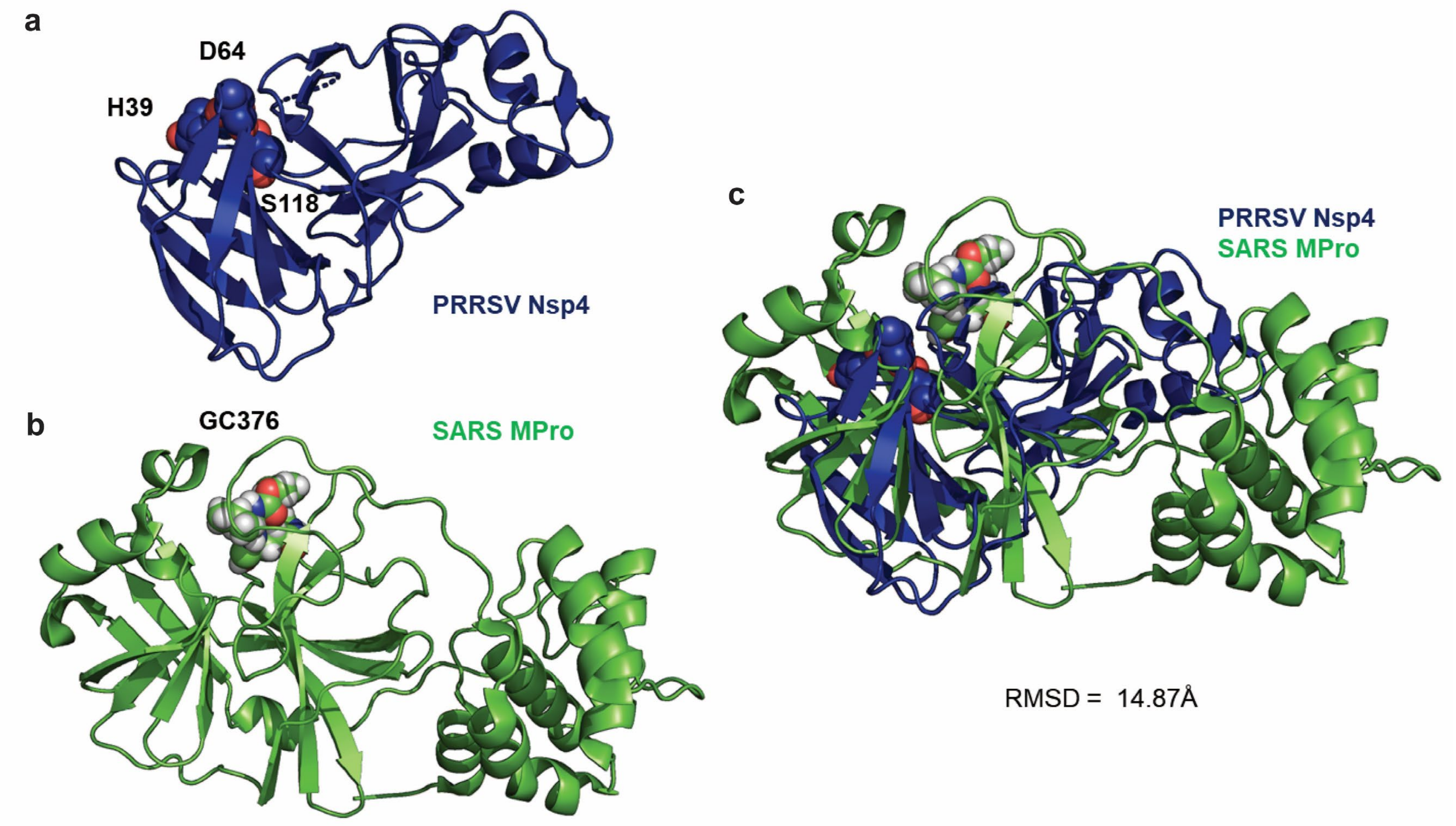

Supplementary Figure 7. Structural comparison of PRRSV nsp4 and SARS-CoV-2 main protease 3CL^{pro} (M^{pro}). a Crystal structure of PRRSV nsp4 (PDB: 3FAN). The catalytic triad—H39, D64, and S118—is highlighted as spheres. **b** Crystal structure of the SARS-CoV-2 main protease (M^{pro}) in complex with the inhibitor GC376 (PDB: 7CB7). **c** Structural alignment of PRRSV nsp4 (blue) and SARS-CoV-2 M^{pro} (green), highlighting conserved features of the catalytic core.

Supplementary Table 1. Primers used for the construction of PRRSV-1 and PRRSV-2 infectious clones and recombinant EGFP virus mutants.

Construct	Fragment	Primer	Sequence (5'-3')
pXH-GD	F1	DK867-F	ACGTATGTTCCCATAGTAACG
		p7175-R	CGGTCCTCATCCCCCCAGGCATTG
	F2	p7033-F	GAGACTAGAGTCCTTGCCGGGTCCA
		DK869-R	ATGCCTGCTATTGTCTTCC
	pBAC-his3	DK871-F	CTGTGCCTTCTAGTTGCC
		DK874-R	GGTGGAGACTTGGAAATCC
pLelystad	F1	Lely-5E-F	CATTGACGTCAATGGGAG
		Lely-5E-R	TTCCAGTGAAGCTTGTCC
	F2	Lely-F1-F	ATGATGTGTAGGGTATTCCCC
		Lely-F1-R	AAGGATCGACTACAGAGAGG
	F3	Lely-F2-F	CTCGAAATGCTTTCTCTGATGG
		Lely-F2-R	TCAACACCTGTGCAGACC
	F4	Lely-F3-F	CTGGACACCCATTGTTGC
		Lely-F3-R	CATGTGATATGCAAGAGCAGC
	F5	Lely-F4-F	CCTGATGCTGGGTTTCAG
		Lely-F4-R	CAACAAGCCTTCATAGGACC
	F6	Lely-F5-F	CCATACTGTTTGGGTTCACC
		Lely-F5-R	TTTTTAATTTCGGTCACATGGTTCCTG
	F7	Lely-3E-F	CAATCAACTGTGCCAGTTGC
		Lely-3E-R	GTCCCATTCGCCATTACC
	pBAC-his3	Lely-BAC-F	ATGGTCCCAGCCTCCT
		Lely-BAC-R	ACGGTTCACTAAACGAGCTC
pGD-1'GFP	F1	DK867-F	ACGTATGTTCCCATAGTAACG
		GD-R1	GTAGCTTTTGACCCAAGC
	F2	GD-F2	GTAACGAGGTTCATCATCGT
		p7175-R	CGGTCCTCATCCCCCCAGGCATTG
	F3	p7033-F	GAGACTAGAGTCCTTGCCGGGTCCA
		Gap-R	CATTTCAATTCAGGCCTA
	F4	GD-ORF1-GFP-TRS6-F	CCCCGGGCCCTGTCATTGAACCAACTTTAGGCCTGAATTGAAATGGTGAGCAAGGGCGAG
		GD-ORF1-GFP-TRS6-R	GCCAATTTTGTCAAAGAGGCTTTGCATGGACCCCATTTCATTGTTCTGCTGAAACTCTGG
	F5	Gap-F	ATGAAATGGGGTCCATGC
		GD-R7	CAAGGTTTACCACTCCCT
	F6	GD-F7	GCTCTGGCTGCGCTGATT
		DK869-R	ATGCCTGCTATTGTCTTCC
	pBAC-his3	DK871-F	CTGTGCCTTCTAGTTGCC
		DK874-R	GGTGGAGACTTGGAAATCC
pGD-7'GFP	F1	DK867-F	ACGTATGTTCCCATAGTAACG

		GD-R1	GTAGCTTTTGACCCAAGC
	F2	GD-F2	GTAACGAGGTTCATCATCGT
		p7175-R	CGGTCCTCATCCCCCCAGGCATTG
	F3	p7033-F	GAGACTAGAGTCCTTGCCGGGTCCA
		GD-R7	CAAGGTTTACCACTCCCT
	F4	GD-F7	GCTCTGGCTGCGCTGATT
		GD-gap-R	TTATGCTGAGGGTGATGCTG
	F5	GD-N-TRS6-GFP-F	CATACTGTGCGTCTGATCCGCGCCACAGCATCACC CTCAGCATGATGGTTCCGCGGCAAC
		GD-N-TRS6-GFP-R	CCAATTCTAACACTGAGGTGCCAAAGAATGCCAGC CCATTACTTGTACAGCTCGTCCATG
	F6	GD-gap-F	TAATGGGCTGGCATTCTTTGGCA
		DK869-R	ATGCCTGCTATTGTCTTCC
	pBAC-his3	DK871-F	CTGTGCCTTCTAGTTGCC
		DK874-R	GGTGGAGACTTGGAAATCC
pLV-1'GFP	F1	Lely-5E-F	CATTGACGTCAATGGGAG
		Lely-F1-R	AAGGATCGACTACAGAGAGG
	F2	Lely-F2-F	CTCGAAATGCTTTCTCTGATGG
		Lely-F2-R	TCAACACCTGTGCAGACC
	F3	Lely-F3-F	CTGGACACCCATTGTTGC
		Lely-F3-R	CATGTGATATGCAAGAGCAGC
	F4	Lely-F4-F	CCTGATGCTGGGTTTCAG
		ORF1-gap-R	CATCACCCCGAATTCACG
	F5	ORF1-GFP-F	ACAGAATTGCAGGTAGAGC
		ORF1-GFP-R	CTAACAAGGAACTCAGTGAAGG
	F6	ORF1-gap-F	ATGCAATGGGGTCACTGT
		Lely-3E-R	GTCCCATTCGCCATTACC
	pBAC-his3	Lely-BAC-F	ATGGTCCCAGCCTCCT
		Lely-BAC-R	ACGGTTCACTAAACGAGCTC
pLV-7'GFP	F1	Lely-5E-F	CATTGACGTCAATGGGAG
		Lely-F1-R	AAGGATCGACTACAGAGAGG
	F2	Lely-F2-F	CTCGAAATGCTTTCTCTGATGG
		Lely-F2-R	TCAACACCTGTGCAGACC
	F3	Lely-F3-F	CTGGACACCCATTGTTGC
		Lely-F3-R	CATGTGATATGCAAGAGCAGC
	F4	Lely-F4-F	CCTGATGCTGGGTTTCAG
		N-gap-R	TTAACTTGCACCCTGACTG
	F5	N-GFP-F	GCTGCCGGTTGCTCATAC
		N-GFP-R	GTGATCGCCCTAATTGAATAGG
	F6	N-gap-F	TAATTTGACAGTCAGGTGAATGG
		Lely-3E-R	GTCCCATTCGCCATTACC
	pBAC-his3	Lely-BAC-F	ATGGTCCCAGCCTCCT
		Lely-BAC-R	ACGGTTCACTAAACGAGCTC

pLV-1'GFP$^{TRS6(GD)}$	F1	Lely-5E-F	CATTGACGTCAATGGGAG
		Lely-F1-R	AAGGATCGACTACAGAGAGG
	F2	Lely-F2-F	CTCGAAATGCTTTCTCTGATGG
		Lely-F2-R	TCAACACCTGTGCAGACC
	F3	Lely-F3-F	CTGGACACCCATTGTTGC
		Lely-F3-R	CATGTGATATGCAAGAGCAGC
	F4	Lely-F4-F	CCTGATGCTGGGTTTCAG
		ORF1-gap-R	CATCACCCCGAATTCACG
	F5	ORF1-GFP-TRS6(2)-F	AACCCCGGCTGCCGCCTGGGCAAGTGCCGTGAATTCGGGGTGATGGTGAGCAAGGGCGAG
		ORF1-GFP-TRS6(2)-R	GAACAGCTGGCTGATTTTACTCCACAGTGACCCCATTGCATTGTTCTGCTGAAACTCTGG
	F6	ORF1-gap-F	ATGCAATGGGGTCACTGT
		Lely-3E-R	GTCCCATTCGCCATTACC
	pBAC-his3	Lely-BAC-F	ATGGTCCCAGCCTCCT
		Lely-BAC-R	ACGGTTCACTAAACGAGCTC
pLV-7'GFP$^{TRS6(GD)}$	F1	Lely-5E-F	CATTGACGTCAATGGGAG
		Lely-F1-R	AAGGATCGACTACAGAGAGG
	F2	Lely-F2-F	CTCGAAATGCTTTCTCTGATGG
		Lely-F2-R	TCAACACCTGTGCAGACC
	F3	Lely-F3-F	CTGGACACCCATTGTTGC
		Lely-F3-R	CATGTGATATGCAAGAGCAGC
	F4	Lely-F4-F	CCTGATGCTGGGTTTCAG
		N-gap-R	TTAACTTGCACCCTGACTG
	F5	N-TRS6(2)-F	CTGATTCGCGTGACTTCTACATCCGCCAGTCAGGGTGCAAGTTGATGGTTCCGCGGCAAC
		N-TRS6(2)-R	GCCACACGCCAATCGCGGCCATTCACCTGACTGTCAAATTACTTGTACAGCTCGTCCATG
	F6	N-gap-F	TAATTTGACAGTCAGGTGAATGG
		Lely-3E-R	GTCCCATTCGCCATTACC
	pBAC-his3	Lely-BAC-F	ATGGTCCCAGCCTCCT
		Lely-BAC-R	ACGGTTCACTAAACGAGCTC
